# Robust and Rapid Air-Borne Odor Tracking without Casting^1,2,3^

**DOI:** 10.1523/ENEURO.0102-15.2015

**Published:** 2015-12-07

**Authors:** Urvashi Bhattacharyya, Upinder Singh Bhalla

**Affiliations:** National Centre for Biological Sciences, TIFR, Bangalore, Karnataka 560065, India

**Keywords:** behavioral strategy, navigation, olfaction, stereo, turbulence

## Abstract

Casting behavior (zigzagging across an odor stream) is common in air/liquid-borne odor tracking in open fields; however, terrestrial odor localization often involves path selection in a familiar environment. To study this, we trained rats to run toward an odor source in a multi-choice olfactory arena with near-laminar airflow. We find that rather than casting, rats run directly toward an odor port, and if this is incorrect, they serially sample other sources. This behavior is consistent and accurate in the presence of perturbations, such as novel odors, background odor, unilateral nostril stitching, and turbulence. We developed a model that predicts that this run-and-scan tracking of air-borne odors is faster than casting, provided there are a small number of targets at known locations. Thus, the combination of best-guess target selection with fallback serial sampling provides a rapid and robust strategy for finding odor sources in familiar surroundings.

## Significance Statement

Our study presents a novel olfactory task making it possible to study air-borne odor tracking under well-controlled airflow conditions, which elicit robust and spontaneous behavioral patterns in rats. The study has numerous implications for the neuroethology of odor**-**guided target selection, and opens up interesting questions about how rats choose between strategies under different conditions that they may encounter in the field. It further sets tight constraints on olfactory sensory processing, both in terms of the sampling time, and in terms of decision-making. We speculate there may also be implications for how animals combine multisensory input for odor**-**guided navigation and target selection.

## Introduction

Odor tracking is an essential capability for survival in many animals, and serves to find and identify food, mates, or predators. Many land animals can navigate toward the odor source using both air-borne and surface**-**borne cues.

Studies show that dogs, humans, and rats zigzag across a surface odor trail during tracking, a strategy known as casting (Gibbons, 1986; Porter et al., 2007; Khan et al., 2012). A similar zigzag strategy is also observed in insects (Vickers, 2000; Willis and Avondet, 2005; Lent et al., 2013), fish (Montgomery et al., 1999; DeBose and Nevitt, 2008), and crustaceans (Weissburg and Zimmer-Faust, 1994; Basil et al., 2000; Vickers, 2000) that have to use odor information dispersed intermittently in fluid media. Apart from such zigzag tracking, animals may also switch between different strategies to compensate for stimulus perturbations, such as different odor gradients (Catania, 2006, 2013; Cardé and Willis, 2008; Reynolds et al., 2009; Gomez-Marin et al., 2010, 2011). In turbulent conditions, vertebrates are known to display phases of tracking that are different from each other in features, such as speed, head movement, or odor sampling rate (Moore et al., 1991; Thesen et al., 1993). Rats also switch rapidly between local and longer-range casting when they lose an odor trail (Khan et al., 2012). Studies in ethologically relevant settings show wider plume sweeping trajectories of animals with unilateral sensor blockage (Webster et al., 2001; Porter et al., 2007; Duistermars et al., 2009; Khan et al., 2012; Catania, 2013). Similarly, animals shift in their orientation and speed to adapt to the presence of masking or distracting background odors, where an animal might have to discriminate between different potential stimuli (Party et al., 2013). They thus learn to deploy a range of behaviors to compensate for perturbations, yet retain accurate odor localization.

Although stereo and casting strategies seem to be effective in many open-field contexts, in many cases there are additional cues. For example, in familiar environments there are likely to be a few known paths leading to food sources, there may be visible targets or the animal may remember the outcomes of past choices. Multisensory, and especially visual input, may also help to change the olfactory task from “where” to “which”. For instance, in the context of multiple-choice elimination problems, the structure of the maze and spatial location of food is important to determine strategy. These factors determine whether the animal eliminates possible targets using a high divergence strategy (ie, choosing locations as far as possible from one trial to next, as in target number 1-4-2-3) or a lateral scanning strategy (going to the nearest target from one just visited, as in target number 1-2-3-4; Poucet et al., 1983; Buhot and Poucet, 1987). It is thus interesting to ask whether strategies other than zigzag “casting” may be suitable in air-borne plume tracking, and how robust these may be to perturbations.

In this study, we developed a near-laminar-flow arena in which free-running rats could track air-borne odors that were well controlled with respect to location, background, and plume dispersal. We find that rather than casting, rats proceed directly to a potential target with a success rate that is much higher than chance, and scan serially across targets if this is wrong. This behavior is robust to a range of conditions, such as odor changes, background odor, unilateral nostril occlusion, and turbulence. We develop a model that shows that this behavior is more efficient than casting for a wide range of conditions.

## Materials and Methods

### Animals

Five male Long–Evan rats, 2 to 3 months old, were used for this study. All of the experimental procedures were approved by the National Centre for Biological Sciences institutional animal ethics committee, in accordance with the guidelines of the Indian National Government and equivalent guidelines of the *Society for Neuroscience.*

### Thermocouple implantation

**Figure 1.**
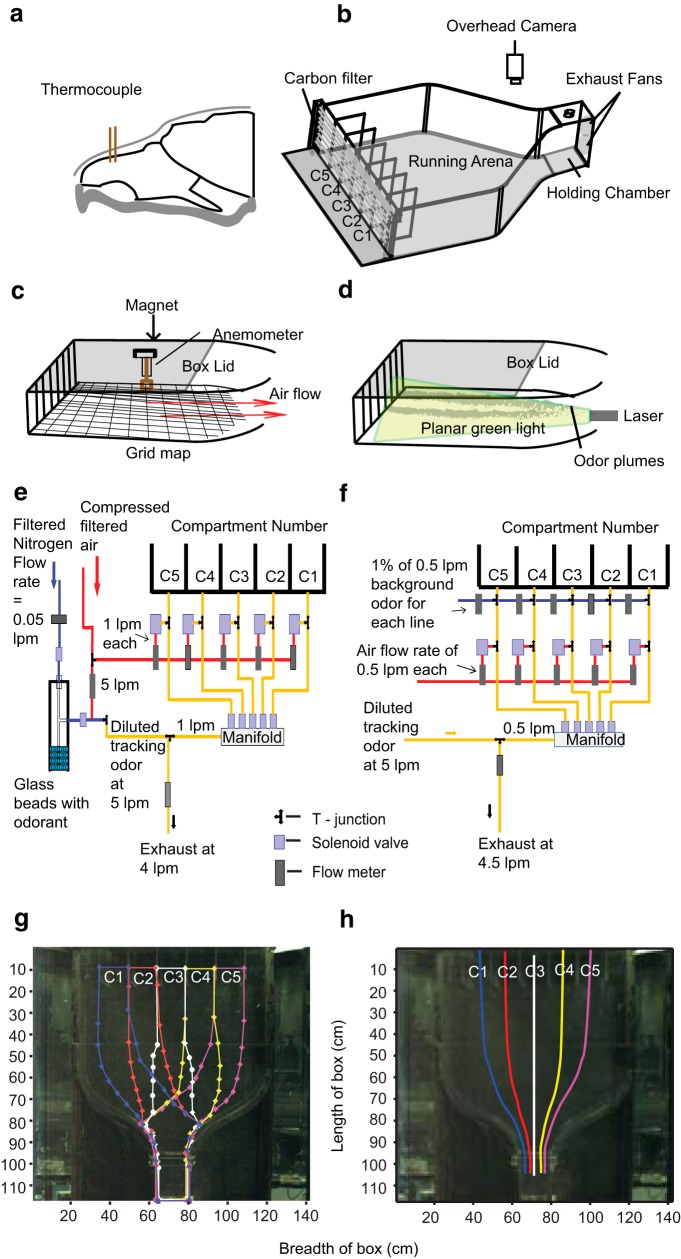
**Thermocouple implant, behavior arena, and methods used for setup standardization. *a***, Schematic of rat’s skull with thermocouple implant. ***b***, Schematic of the behavior box with overhead camera. Compartments are indicated from C1 to C5. There are dummy compartments between C1 and the wall, and C5 and the wall, to keep the odor flow away from the walls. ***c***, Cross section of the behavior box and placement of anemometer at grid lines for airflow measurement. ***d***, Odor plume visualization using planar green laser light. ***e***, Olfactometer design in baseline IAA tracking experiments. ***f***, Olfactometer design with background odor experiments. ***g***, Specified boundary regions for defining trial outcome and strategy, color coded for each compartment. ***h***, Projected odor path from each compartment used for calculation for deviation of trajectory from odor trail, color coded from C1–C5.

To monitor respiration during the behavior task, rats were implanted with thermocouple (PhysiTemp, Copper-Constantan, insulated) in their nasal cavity (Fig. 1*a*). Skull holes were drilled at ∼5 mm anterior to the nasal suture and ∼2 mm lateral to the midline. The thermocouple wire was soldered onto a connector board, which was cemented using six skull screws. For all surgical procedures, rats were anaesthetized with 4% halothane and anesthesia was maintained with 1–2.5% halothane. For the nostril occlusion experiments, one of the nostrils was stitched shut with one or two stitches while the rat was under anesthesia. The integrity of the stitches was checked after stitching and before the training session. Removal of stitches was also done under anesthesia. Postsurgical care involved cleaning the suture site with iodine solution, followed by application of neomycin sulphate antibiotic powder over the wound. Rats were given the general analgesic Dolo SUS (paracetamol, 100 mg/kg, Micro Laboratories) for 3 d and allowed to recover for another 4 d before training was initiated.

### Training box

The behavior arena was custom designed with a funnel-shaped cross-section. Its dimensions were 114.3 cm (l) × 88.9 cm (b) × 25.4 cm (h), with a curving angle of 44° centered in the middle (Fig. 1*b*). The broader opening of the funnel was divided into seven compartments, each of dimension 12.7 cm (b) × 25.4 cm (h) and extending 15.24 cm (l) into the box. The central five compartments were used to deliver odor. Rats were placed in the narrow opening of the funnel [22.86 cm (l) × 15.24 cm (b) × 25.4 cm (h)] that served as both the holding chamber and the reward delivery location between trials. Two circular exhaust fans (11.43 cm diameter each) were fixed on the wall of the holding chamber to provide air suction. All the experiments, except those requiring turbulent airflows, were conducted with the broader end covered using an “activated carbon filter” (5 mm) sandwiched between fine steel mesh.

### Airflow velocity measurement

Anemometer measurements were conducted for the running arena, starting from 21 cm ahead of the odor source compartments (*Y* = 21) and ending 6 cm before the holding chamber (*Y* = 81 cm). The running arena was sampled in a grid of dimensions 1 cm (*x*-axis; range: −44,44 at maximum width) by 6 cm (*y*-axis; range: 21, 81). A hot wire anemometer (Kurz instruments 490-IS-M) was suspended in the closed behavior box using magnets at each intersection of the grid points (Fig. 1*c*). The tip of the anemometer filament was positioned at ∼5 cm from the base. Data were collected using a Measurement Computing data acquisition card (MCC DAQ PCI-6023) for 10 s at a sample rate of 200/s. The anemometer voltage readings were calibrated against airflow measured using the provided meter as well as against another precalibrated anemometer. This curve was fitted using excel to the following equation:

velocity=0.0817∗exp(12.795×V)

Where velocity is in m/s and *V* is voltage out. This conversion equation was used to estimate the airflow rate from anemometer samples.

### Smoke plume visualization

Smoke sticks were placed at the level of the odor source tube for each compartment (∼3 cm) and visualized using a planar laser-induced scattering technique. The green laser pointer (<5 mW) was placed at the holding chamber. The light passed through a cylindrical lens to form a sheet of ∼2 mm thickness (Fig. 1*d*). Video was captured using either a SONY Handycam DCR-SR300E (Movies 1, 4) or iBall c12.0 webcam (see Fig. 13*a*,*b*). The images collected were stacked and contrast enhanced in ImageJ for depicting the path of the smoke plume.

### Odor stimulus delivery

A custom-built air dilution olfactometer was used for odor delivery (Fig. 1*e*). Nitrogen at a constant flow rate of 0.05 l/min controlled using mass flow controller (Alicat Scientific) was passed through a glass bead bubbler containing liquid odor. The odorized nitrogen stream was diluted with 4.95 l/min of clean, humidified air to give a diluted concentration of 1% odor [isoamyl acetate (IAA); Cineole, Limonene Tracking]. Diluted odorized airstream at 1 l/min from a randomly selected odor port and 1 l/min of plain humidified air from the remaining four ports was introduced into the behavior box using relay controlled solenoid valves. Olfactometer design was changed for the background odor introduction experiments (Fig. 1*f*) to maintain the overall flow rate of the odor mix at 1 l/min. Tracking odor (1% at 0.5 l/min) was introduced along with the background odor (1% at 0.5 l/min) in the selected odor port and clean humidified air (0.5 l/min) with background odor (1% at 0.5 l/min) was introduced from the remaining four ports. The effective concentration of the odors in the stream was thus reduced to one-half of that used in baseline experiments. The olfactometer and water delivery were controlled using a custom written Microsoft Visual C# program. Odor-source compartment selection for each trial was randomly generated. Solenoids and their on/off state visualizing light emitting diodes were controlled using an R16 relay board.

### Olfactometer calibration and odor measurement in behavior box

The olfactometer was calibrated using a photo ionization detector (PID; mini PID, Aurora Scientific). The probe was sequentially placed in front of all the odor source outlets under both normal and background odor conditions to verify odor concentration and delivery time. The PID map for odor in the box was generated by placing the probe at *X* = 1 cm along the breadth and *Y* = ((15, 33, 45, 57, 69, 81) cm along the length of the box, where *Y* = 0 marks the entrance of the odor compartments. We were only able to detect the presence and absence of odor at a given position using this technique. Calibration was done using isoamyl acetate as the tracking odor with four repetitions of odor delivery in two sessions each at a given location.

### Wireless transmission

The thermocouple signals were obtained using a 15 channel wireless transmitter (Triangle Biosystems) transmitting signals at 100 k samples/s. Signals were differentially digitized at 200 Hz and saved to disk using Measurement Computing DAQ (PCI-6023) with custom written MATLAB (Simulink, MathWorks) program.

### Training procedure for navigation task

Implanted rats were trained to shuttle back and forth between the odor port/compartment and the water reward/holding chamber. The training was established in four modules:

*Habituation to behavior box* (*2 d*): rats were placed in the behavior arena for 10 min each, for box exploration and habituation.
*Shuttle to water reward port* (*2 d*): rats were trained to shuttle back and forth between the arena and the holding chamber for water reward, as well as initiation of the next trial. Each session lasted for 10 min.
*Associate water reward with odor localization* (*∼7 d*): odor was introduced in the box at this stage. Rats were trained to identify the correct odor port and shuttle back to receive water reward at the holding chamber. The rats were trained for 20 min each session until they learned to self-initiate each trial.
*Achieve 80%*: this was an extension to Module 3 where rats were required to correctly locate the odor port and shuttle back to water reward with an accuracy of 80% or higher.

## Experimental tasks and their order

*Tracking in laminar airflow velocity*: the basic training task was performed with thermocouple-implanted rats under laminar airflow conditions. One liter per minute of diluted odor (IAA, 1%) was introduced from randomly selected odor port until rats learned to locate the correct odor port with >80% accuracy. This training session lasted for ∼40 d.
*Task generalization*: to verify that the rat’s response was not specific to IAA, cineole (1 l/min at 1%) and later limonene (1 l/min at 1%) were introduced as tracking odors to confirm task repeatability and task generalization. The rats tracked cineole and limonene for 6 and 4 d, respectively.
*Tracking in the presence of novel background odor*: rats were assigned the task to track one odor (limonene, 0.5%, 1 l/min) in the presence of an untrained (linalool, menthone) odor as background conditions (0.5%, 1 l/min) flow. The protocol was as follows: (1) limonene + air (3 d), (2) limonene + linalool (2 d), (3) limonene + air (1 d), and (4) limonene + menthone (2 d).
*Tracking under non-stereo conditions*: four rats were used for this task. Unilateral nostril stitch was performed on either the left (2 rats) or the right nostril (2 rats) on the third day of baseline tracking. Rats tracked odor with nostril stitch on the fourth day, followed by 2 d of recovery.
*Tracking in the presence of familiar background odor*: same task as in 3, except with IAA as background odor. Protocol was as follows: (1) limonene + air (2 d) and (2) limonene + IAA (5 d).
*Turbulent airflow conditions*: the carbon filter mesh was removed for this task, such that the airstream eddies could not be broken down into streamlined flow. Four rats were assigned to track limonene (1% at 1 l/min flow rate) coming from a randomly selected port.

### Video imaging and analysis

The behavior was recorded using a high-speed camera (Silicon Imaging, SI-1920) and XCAP imaging software at 60 fps for 30-min-long session per rat each experimental day. Frames (70,000–120,000) at an area of interest of 792 × 960 pixels were captured per rat depending upon the duration of each session. Individual LEDs corresponding to olfactometer switch, water reward, synchronization of thermocouple recording, and activation of each compartment source were also placed in the field-of-view. Video files were converted to .AVI format and compressed using Virtual Dub for further analysis. Wireless headstage had blue, green, and red LEDs fixed on top for tracking. A custom written MATLAB program identified the position of these LEDs for each frame using a threshold for each color. Missing points were obtained using interpolation of the track using the cubic spline method in MATLAB. Additionally, each frame was time stamped during recording by the video acquisition software (EPIX-XCAP) for time synchronization with thermocouple data. Further analysis with data from position coordinates was done in MATLAB, Python, and R.

### Trial outcome and strategy classification

For a given trial, the start and end points of the trial were respectively fixed at the frames where the rat emerged from, and returned to the holding chamber. Forward path was defined as the trajectory starting from the beginning of a trial until the first odor compartment entry, whereas the return path of a rat was defined as the trajectory from the last compartment exit until reaching the holding and reward chamber. Three main criteria were used for a trial to be classified as direct: (1) a single odor compartment entry in the forward path, (2) 85% of the forward track lay within a predefined region for a compartmental source (Fig. 1*g*), and (3) a relatively linear path with a maximum absolute deviation from the projected odor path ≤22 cm (Fig. 1*h*). For the purposes of delivering enhanced reward for direct trials, an online analysis was done using only the first of these criteria. The subcategorization of the direct trials into “straight” and “offset” was again done on the basis of absolute maximum deviation from projected odor stream. Trials with deviation up to 15 cm were termed as straight and those with deviation from 15 to 22 cm were termed as offset. All paths having entries into multiple target compartments or large lateral scans were clubbed under the serial strategy of target selection. For the outcome to be classified as correct, the last compartment exit had to be from the odor source compartment.

Zigzag trials were identified by visual inspection of forward tracks. From data based on smoke plumes, an outline of ±2.5 cm from the projected odor trail was taken as the maximum width of the plume. We used a criterion of sharp changes in direction and at least two crossings of the odor stream over a length of 80 cm from the holding chamber to the beginning of the odor compartment to classify the behavior as casting.

### Root mean square deviation calculation

Fig. 1*h* shows projected odor path for each compartment used to calculate root mean square (RMS) deviation. These central odor lines were used to calculate the deviation of rats’ trajectory along *x*-axis. Subsequently, root mean square deviation for the entire trajectory in the forward direction was calculated.

### Sniffing frequency calculation

The radio-recorded thermocouple signal was first filtered using a bandpass filter (MATLAB ellip with cutoffs L1 = 1, H1 = 0.5, L2 = 20, and H2 = 30, all values in Hz), and mean subtracted. It was then subjected to Fourier analysis using the numpy.fft.rfft function. As the original signal was noisy due to mechanical transients and transmitter noise, we applied the following criteria in order to select for acceptable recordings. We required that the frequency peaks obtained on the left and right channels were within 1.5 Hz of each other, and that the frequency was at least 3 Hz in the forward direction and 2 Hz in the return direction. We also required that the threshold for the forward-direction frequency peak height was >0.007, and 0.009 for reverse. Finally, we required that the ratio of the second-highest frequency peak to the highest peak was no more than 0.6. These criteria were developed based on visual inspection of >100 trial waveforms, encoded in Python, and applied to all trials. Approximately 15% of trials cleared these criteria and were used for respiration analysis.

### Sniff timing estimation

We took the same filtered thermocouple signal as above. We first selected only waveforms where the signal had a SD >0.015 V. We then required that the waveform should rise monotonically from −0.01 to >0.01 V, and picked the zero-crossing point as the time of inhalation. Having done this for both left and right respiration channels, we combined the signals with the further requirement that if both channels reported a putative simultaneous inhalation it should be within 15 ms of each other. Finally, we eliminated cases where the time since last inhalation was <66 ms (corresponding to 15 Hz sniffing). These classification criteria were developed, as above, based on visual inspection of >100 trial waveforms. The classification was encoded in Python and applied to all trials. Due to mechanical and transmission noise, only a small fraction of inhalation events cleared these criteria. These inhalation points were used to analyze sniff-triggered course changes.

### Calculation of sniff-triggered course changes

To measure changes in orientation of rats post-sniff, the time-point of inhalation during forward track was marked as Frame 0 (Time 0; Fig. 14). Displacement of rats along the *x*-axis (dx) per frame (dt) for the next 40 frames (∼680 ms) was calculated and averaged for all post-sniff periods for a given session. As a control, we computed the displacement as above, but triggered respectively from each of the frames from sniff−4 to sniff+4. We averaged these displacement estimates to obtain control displacements. At 60 frames per second, these 9 frames span approximately 133 ms, which is approximately the same as the average sniff duration. As mentioned above, the inhalation timing measurements cleared criteria in only a fraction of recordings. Overall, we were able to use 46 recording sessions, which had sufficiently clean sniff timings.

### Statistical analysis

The non-normal distributions were tested for significance in MATLAB using nonparametric test, Kruskal–Wallis (KW) ANOVA followed by Tukey HSD for multiple comparisons (multcompare) at 5% significance values. These included speed, RMS deviations, and time taken for direct and serial tracking in each experimental module. Box whisker plots in Figures 4, 10, 11, 12, and 13 represent the 25^th^ percentile (bottom edge) and 75^th^ percentile (top edge) of the data. The median is represented by the gray line, most extreme data points by whiskers and outliers marked individually with gray colored crosses. All error bars in line graphs represent the SEM for Nd (number of direct trials) or Ns (number of serial trials). Data for all rats were pooled to obtain the values of mean and SEM across days (line graphs) and for an entire block of experimental module (box whisker plots).

To compute whether first entries for the odor compartment were significantly different from a chance flat distribution, we carried out χ^2^ test with the observed frequency of first entries across diagonals (Fig. 3) and expected frequency (total number of trials/25) for each rat using MS Excel “chitest”.

For side compartment preference calculation, Two tailed Student’s *t* test for each rat was performed in MATLAB between the number of first entries in off diagonal (*k* ± 1) non-odor compartments (8 bins) versus the remaining non-odor compartments (*k* ± 2, *k* ± 3, and *k* ± 4, 12 bins; Fig.3).

Instantaneous speed and deviation for each forward track position until the first entries were pooled from all baseline days for all rats. Subsequently, the deviation and speeds were averaged for every 1 cm increment from holding chamber until odor source. Paired sample *t* test was used to test for significance between averaged speeds in direct and serial tracks.

To test significant differences within post-sniff displacement, and between post-sniff versus control displacement, *Z* test, followed by Bonferroni correction (0.05/46, α = 0.001) was performed for all sniff samples.

### Model parameters

We estimated tScan in two ways. First, we assumed that the rat remembered which compartments it had tested, including the first entry. Thus, the expectation number of compartments to test in the case of a wrong entry was 2. Given that there was a 3 s difference between entry into the correct compartment for serial versus direct trials (Fig. 4*g*), we obtained the estimated tScan = 1.5 s. The second estimate of tScan came simply from analyzing the videos where the rat sampled multiple compartments. We manually analyzed five to six trials per rat, totaling 55 entries in different compartments. We obtained an estimate of 1.4 ± 0.07 s for tScan.

## Results

### Near-laminar airflow in behavior box

**Figure 2.**
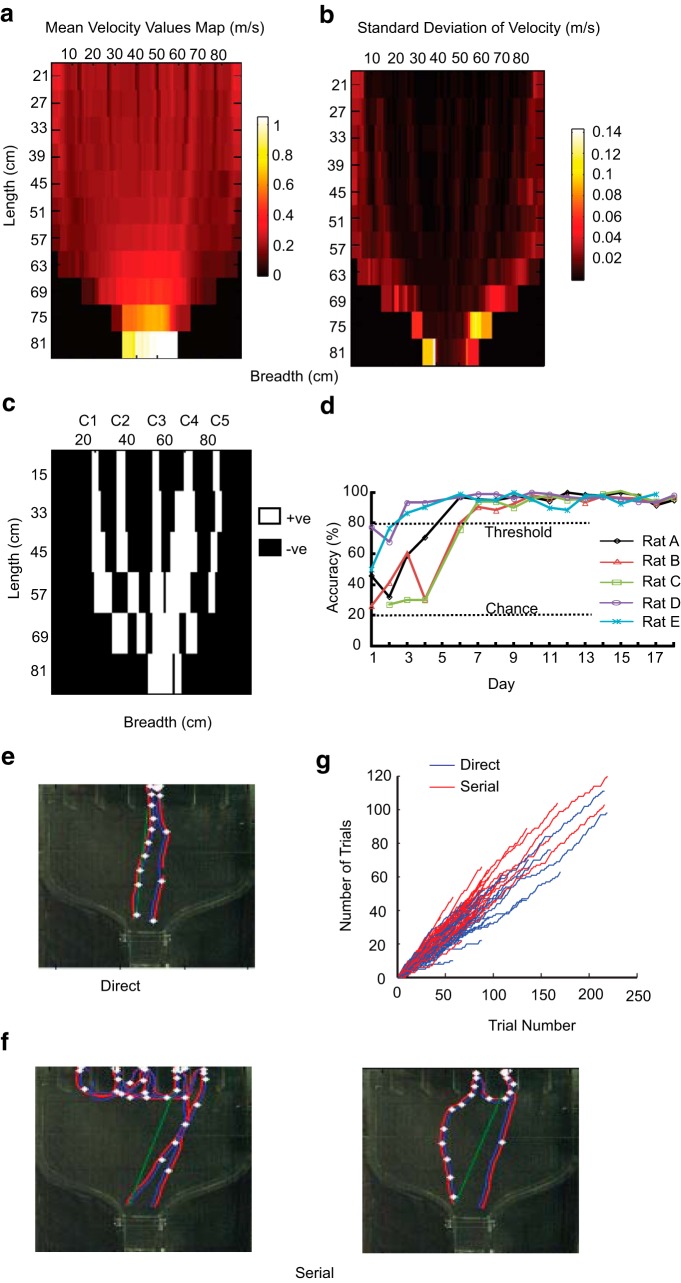
**Near laminar airflow in behavior box. *a***, Heat map of the mean airflow velocity in the box as measured by anemometer. ***b***, Heat map of the SD of airflow velocity in the box. Airflow was very stable except near the walls. ***c***, Readout of IAA detection in the box using a PID for the five central compartments. White regions represent IAA presence. ***d***, Learning curve for correct odor source location for all five rats. Chance accuracy level is 20% and criterion level for accuracy is 80%. ***e*, *f*,** Different tracking strategies based on trajectory of odor source location. ***e***, Direct trial, whereas ***f*** represents examples of serial (left) or lateral scan (right) to find the correct odor compartment. Red and blue lines are the tracking LED positions. The red LED was present on the left side of the rat. White dots represent estimated inhalation points in the sniff cycle. Green lines project from the holding chamber toward the correct odor compartment. ***g***, Cumulative distribution of direct and serial trials as a function of trial number, pooled across all baseline days and all rats.

To monitor air-borne odor-guided behavior, we designed a multi-choice behavior arena similar to a closed air tunnel with near laminar airflow. Figure 1*b* shows a side view of the funnel-shaped behavior box that includes a holding chamber for the rat, a running arena, and five different compartments as odor sources (see Materials and Methods). To measure airflow inside the box, we used a hot-wire anemometer suspended inside the box through the lid, using a pair of magnets, to minimize perturbations to the airflow (Fig. 1*c*). The anemometer hung at 4–5 cm above the box floor, and was moved every 1 cm along the breadth and 6 cm along the length of the box in order to sample flow (see Materials and Methods). As measured by the anemometer, airflow in the behavior box was nearly laminar (∼0.3 m/s air velocity; Fig. 2*a*), with very low fluctuations of air velocity (Fig. 2*b*) throughout the tracking arena. Expectedly, air velocity was slightly higher, with larger SD in the region of the box which narrowed near the holding chamber. Maximum velocity was reached at the holding chamber area (∼1 m/s), where exhaust fans pulled air out of the chamber. To visualize and measure odor plume dispersal under these conditions, we used two procedures. In the first procedure, smoke plumes were introduced at approximately the same position as the odor source, ie, ∼3 cm above the floor of the box. These plumes were visualized with a planar laser light sheet (Fig. 1*d*). In the second procedure, we placed a PID at different positions in the box and used olfactometer stimulus delivery to check for presence or absence of odor at a given spot (see Materials and Methods). Both of these methods gave comparable results for trajectory and width of the odor stream. Using the videos, we ascertained that odor streams from different compartments were near laminar, and that plume structures were confined in a narrow (∼4 cm) band (Movie 1). Smoke plume videos and measurements of IAA presence obtained using the PID (Fig. 2*c*) both showed distinct, non-overlapping odor streams from different compartment sources in the running arena. Some overlap was observed at the beginning of the holding chamber for adjacent compartments (C1–C2, C2–C3, C3–C4, and C4–C5). Thus the airflow in the behavior arena was nearly laminar and narrow (∼4 cm). The airflow was also found to be consistent upon repeated visualization using smoke plumes.

Movie 1. **Laminar airflow**. Top view of behavior box with laminar flow conditions. Smoke plumes are released into the box from compartment 5 and visualized using green laser light. Video recorded at 25 Hz. 10.1523/ENEURO.0102-15.2015.video.1

### Rats advance directly to a target, and scan serially if it is wrong

All five rats learned to identify the odor source accurately over the course of 18 d, as measured by the odorized compartment being the last compartment visited before returning for the water reward (Fig. 2*d*; see Materials and Methods for trial outcome criteria). The accuracy after training was over 90%. Two main trajectories of odor source location were observed: “direct” and “serial” (Fig. 2*e*,*f*; Movies 2, 3; recorded at 60 Hz, playback at 25 Hz). Automatic classification of these paths was based on trajectories of the rat (see Materials and Methods). As the direct path seemed to use odor tracking, we sought to obtain greater numbers of direct trials by giving the rats a twofold higher water reward for direct trials. Despite this, each animal maintained a consistent, relatively high rate of serial trajectories. To examine whether casting behavior contributed to direct tracking, we performed automatic offline classification of direct trials into two subcategories: straight and offset (see Materials and Methods). We found that overall, ∼45% of trials were direct/straight, ∼7% were direct/offset, and ∼48% were serial. The direct trials were examined visually for typical features of casting behavior (see Materials and Methods for criteria of selection). We found that only 8% of the total direct trials were classified as zigzag.

Movie 2. **Direct tracking**. A rat implanted with thermocouple is tracking odor source by running directly toward it. Odor compartment 1. Video recorded at 60 Hz, playback at 30 Hz. 10.1523/ENEURO.0102-15.2015.video.2

Movie 3. **Serial tracking**. A rat implanted with thermocouple is tracking odor source by lateral or serial scans. Odor compartment 3. Video recorded at 60 Hz, playback at 30 Hz. 10.1523/ENEURO.0102-15.2015.video.3

Thus, the first pass analysis of rat trajectories showed that even after extensive training they persistently made a high fraction of errors in their choice of first compartment. The correct trials were mostly in a direct line to the target, and zigzag casting behavior was infrequent.

### Rats show a bias toward a subset of compartments but track odor well within this subset

We next sought to verify whether direct runs were a result of a random selection of compartments or guided by odor. Because the selectivity for direct trials was found to be independent of trial number (Fig. 2*g*), we ruled out motivation as a selection factor for direct versus serial tracking.

Compartment bias was assessed by considering the distribution of first-entry into the five compartments. If the choice was random, then the first entries should be equally distributed among the five choices. Alternatively, if there were a preference toward a single compartment this should show up in a strong bias of first-entry choices. The ability to track odors was measured in two ways. First, we asked whether the odorized compartment was the first visited. Second, we asked whether the most common errors were when the first entry was in the compartment adjacent to the correct compartment.

**Figure 3.**
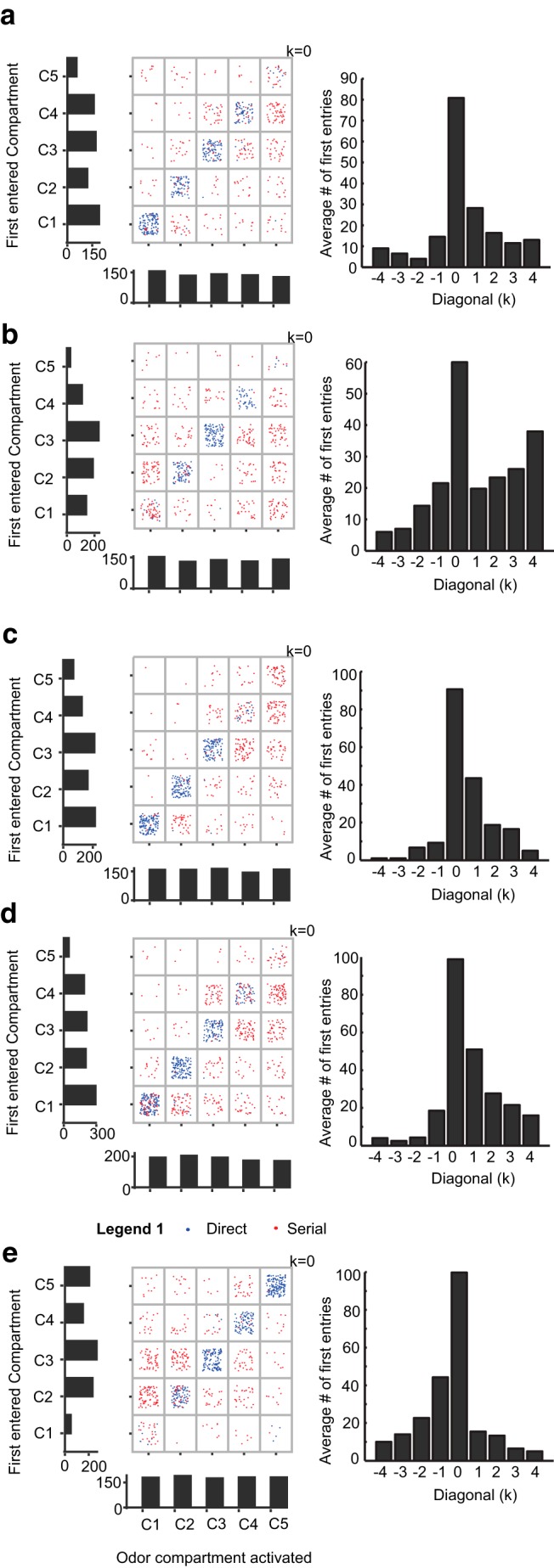
**Odor port versus first compartment choice distributions. *a*–*e***, Data from five rats. Scatter plots show first entry against odor compartment, for each trial. Blue dots indicate direct trials, and these are on the diagonals. Red dots represent scan trials. Data is pooled for 10 d. Vertical histograms show distribution of first compartment entry. These show a preference for a subset of compartments. Horizontal histograms below the scatter plot are distribution of odor delivery compartments. As expected, these are flat distributions. Right column, Histograms of average bin counts along diagonals, with zero bin showing diagonal line, ± 1 bins showing bins adjacent to diagonal, and so on. The central bin is much larger than the others, in all cases. The ±1 bins are larger than the other off-diagonal bins in some cases.

To assess these factors, we plotted a scatter grid of first compartments visited, against odor compartment (Fig. 3). From this dataset, we extracted the distribution of odor compartment activation (bottom histograms) and the distribution of first entries (left histograms). We anticipated that rats might prefer to track the walls of the arena when doing serial trials and that they might do more direct trials at the beginning of the session when they were more motivated. Instead, we found that each rat had a characteristic preference for three or four adjacent compartments (Fig. 3, left histograms).

**Figure 4.**
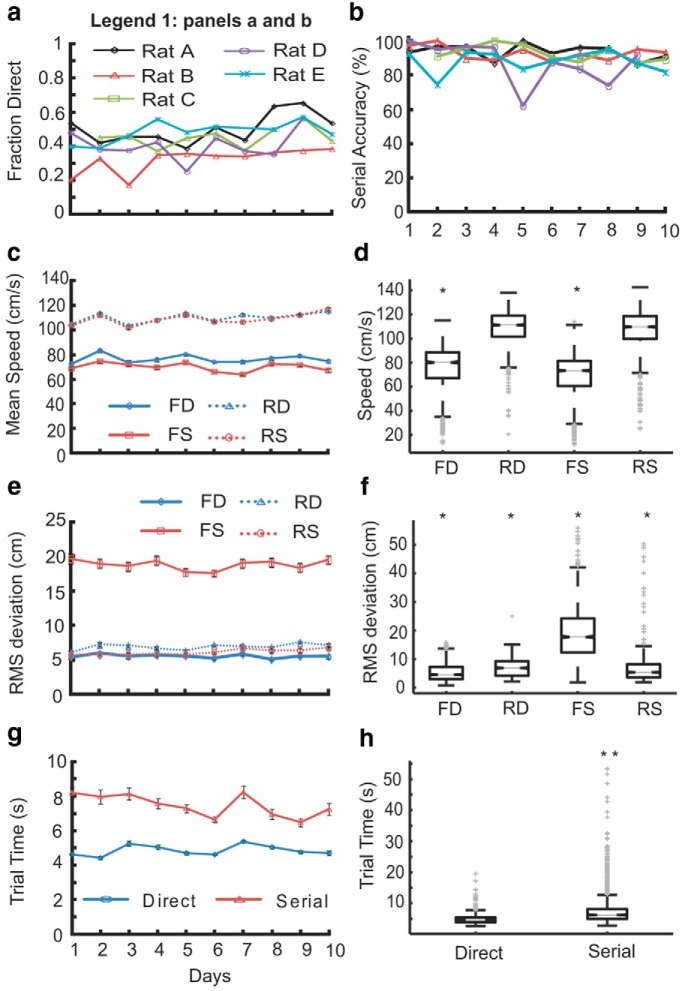
**Baseline measures of tracking strategies of trained rats.** Data shown for days when accuracy was consistently higher than 80%. ***a***, Fraction of direct trials for each rat. ***b***, Percentage of correct serial trials out of total number of serial trials. ***c***, Average speeds of all rats across days for direct (D, blue) and serial (S, red) trials in the forward (F, solid lines) and reverse (R, dashed lines) direction. ***d***, Pooled data across all rats for all days. Forward speeds (FD, FS) are significantly different from each other and from return speeds (RD, RS). No significant difference found between return speeds (*p = 0, KW Tukey HSD test. ***e***, RMS deviation of rat trajectory from an extrapolated odor path for direct (D, blue) and serial (S, red) trials. Solid lines represent forward direction (F) and dashed lines represent return direction (R). All RMS values are statistically significantly different from each other. ***f***, Pooled data of all five rats from all 10 d; *p = 0, KW followed by Tukey HSD test. ***g***, Total time taken for direct (blue) and serial (red) trials. Trial time is significantly different for the two classified groups (KW Tukey HSD test, **p < 10^−10^; ***h***). Number of direct trials: *N*d, = 1851; number of serial trials: *N*s, =2150. Legend 1 for ***a*, *b***. All error bars for ***c***, ***e***, ***g***, are in SEM. ***d***, ***f***, ***h*,** Box whisker plots, representing the median (gray line), 25^th^ percentile (bottom edge of box), 75^th^ percentile (top edge of box), most extreme data points (whiskers), and outliers marked individually (gray crosses).

How accurate was rat tracking by odor? Assuming that rats do indeed prefer to go directly to the odorized compartment, perfect tracking should yield points only along the diagonal. We found that the scatter plot did have a higher density of points along the diagonal, but compartment preferences were also clearly visible as horizontal bands (Fig. 3*b*,*c*). We confirmed that histograms of compartment preference were significantly different from a flat distribution (χ^2^, *p* < 10^−81^ all rats). We also estimated the fraction of correct trials with direct tracking, averaged over all compartments as a function of time (Fig. 4*a*). If compartment entries were independent of odor, one would expect this fraction to be 0.2 (as there were 5 compartments). Instead, the fraction varied between 0.4 and 0.6. Another qualitative trend was that the flanks of the diagonal were also over-represented (Fig. 3, right column histograms, off diagonal *k* ±1), suggesting that when rats made an error, it was mostly to adjacent compartments. We found that the off-diagonal flank visits (*k* ±1, 8 bins) were weakly significant in three rats over visits in the remaining non-odor compartments (*k* ± 2, *k* ± 3, and *k* ± 4, 12 bins; *p* = 0.026, 0.019, 0.036, two-tailed Student’s *t* test).

Of all the serial trials, 43% were correct upon second entry; 31% were correct when the first entry was adjacent and ∼11% were correct when first entries were not adjacent to the correct compartment. Notwithstanding these general trends, it was clear that each rat showed idiosyncratic preferences for a subset of compartments, mostly centered around the middle compartment. In trials where odor was delivered to these compartments the odor-guided tracking was significantly higher than chance.

### Rats run slower but sniff faster during tracking

We next tested whether first-compartment targeting errors were due to reduced monitoring of the odor environment. To do this, we asked whether rats ran and sampled differently when tracking, compared with returning to the reward chamber. We also compared running speed and sampling between direct and serial trajectories.

We observed that rats ran significantly slower (Fig. 4*c*) in the forward direction compared with the return direction [data shown for correct trials: forward direct (FD) = 76.23 ± 0.4 cm/s; return direct (RD) = 109.6 ± 0.3 cm/s; forward serial (FS) = 69.4 ± 0.3 cm/s; return serial (RS) = 108.2 ± 0.3 cm/s]. The forward speeds for direct and serial trials were significantly different from each other as well (Fig. 4*d*; *p* = 0, KW Tukey HSD test).

**Figure 5.**
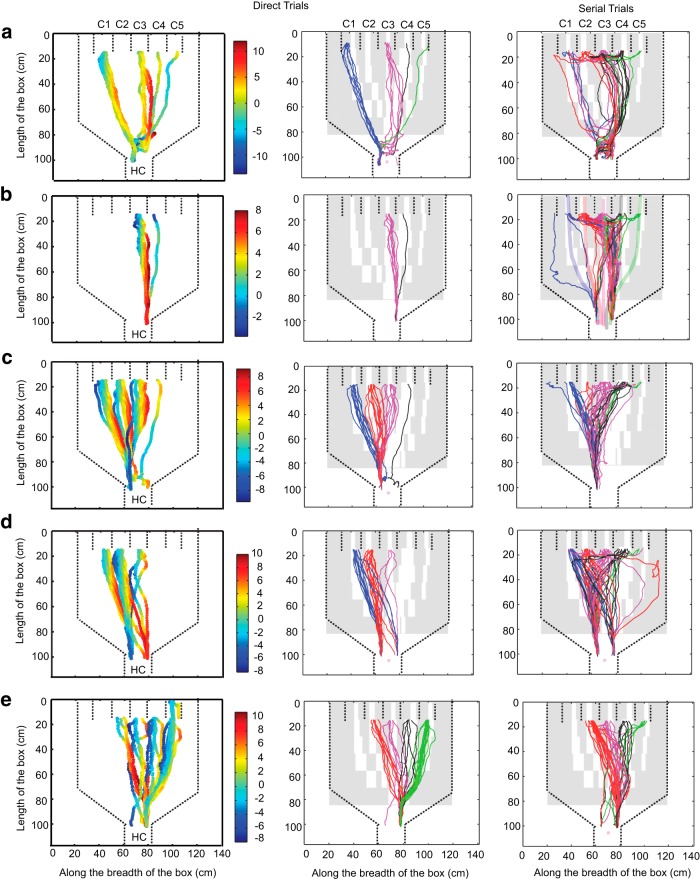
**Instantaneous deviation of trajectory from odor path for each rat. *a*–*e***, Deviation plotted for forward tracks only. Left column panels are deviations for direct trials for 1 d. Deviation values are plotted as color scale (cm). Central and right columns show examples of tracks for direct and serial trials respectively, overlaid on observed odor plumes from Figure 2*c*. Tracks are color-coded for each compartment: C1, blue; C2, red; C3, pink; C4, black; and C5, green. . Dashed lines represent boundaries of the box and the compartments. HC, Holding chamber; C1 to C5, compartment numbers 1 to 5.

Serial tracking was clearly less efficient than direct (Figs. 4*b*–*h*, 16*a*), and had some attributes of exploration. It differed from the direct trials in features such as speed, deviation from odor path and total time to finish trial. The instantaneous deviation of the trajectory from the odor path in direct and serial trials is shown in Figure 5 (*a*–*e*). Left column shows the deviation values for direct trials, whereas the central and right column shows the paths overlaid on observed plume trajectories using PID. The RMS deviation of each rat’s trajectory from the odor path was between 4 and 6 cm (all rats pooled mean = 5.5 cm, ± 0.07 cm, *N*d = 1851) for direct trials and between 15 to 20 cm (all rats pooled mean = 18.6 cm, ± 0.18, *N*s = 2150) for serial trials (Fig. 4*e*). Pooled across all days for all the rats, the averaged RMS values in forward and return paths for both the direct and serial trials were significantly different from each other (*p* = 0, KW Tukey HSD test; Fig. 4*f*). Direct trials were also shorter in duration (*p* < 10^−10^, KW Tukey HSD test) with a mean time of ∼4.8 ± 0.03 s across all rats (Fig. 4*g*,*h*). In contrast, rats took much longer time to finish serial trials (∼7.4 ± 0.09 s).

**Figure 6.**
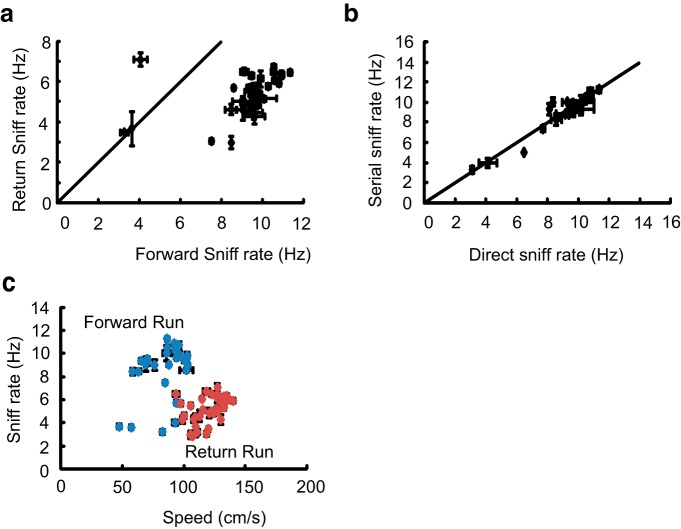
**Rats sniff actively when going forward toward the odor compartment. *a***, Scatter of sniff rate in forward path versus return path averaged over the entire dataset, all rats. Line is for equal rates. Forward rate is almost always faster than return. ***b***, Sniff rate for direct and serial trials is the same. Each point is mean of rate in serial trials versus direct trials for a given rat on a given day. Line is for equal rates. ***c***, Average sniff rate plotted against average running speeds. Blue dots are forward direction and red dots are return. These form distinct clusters. Forward sniff is faster, and run is slower, than return.

**Figure 7.**
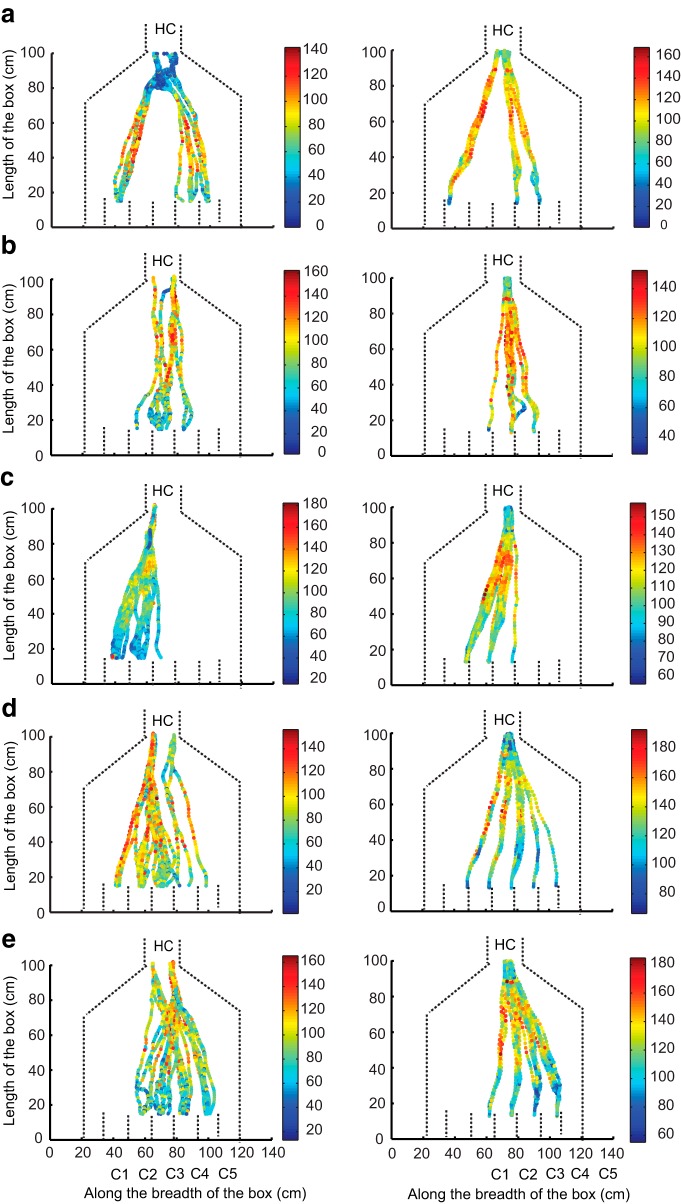
**Instantaneous speed for direct trials plotted for each rat. *a*–*e***, Left column shows the speeds plotted for forward direction. Right column shows the speeds in reverse direction. The color bars show the values of the speeds (in cm/s). *x*-Axis is the breadth of the box (in cm) and *y*-axis is length of the box (in cm). Dashed lines represent boundaries of the box and the compartments. HC, Holding Chamber; C1 to C5, compartment numbers 1 to 5. The plots show multiple trials for a single day.

**Figure 8.**
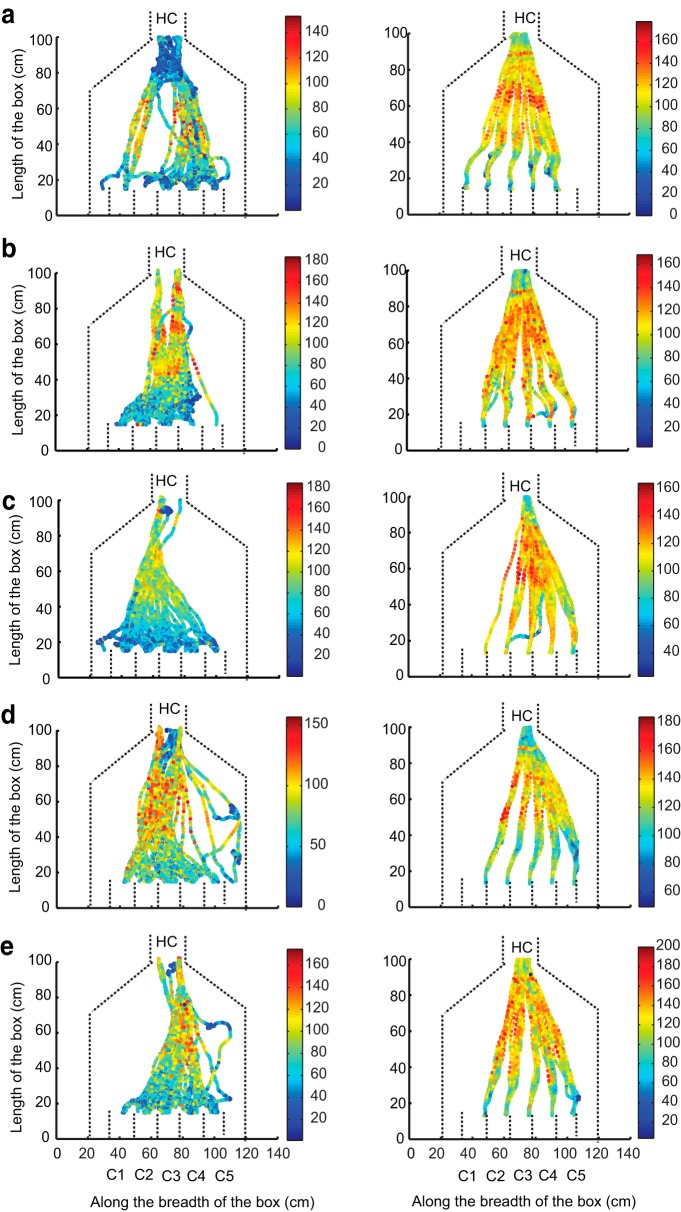
**Instantaneous speed for serial trials plotted for each rat. *a*–*e***, Left column shows the speeds plotted for forward direction. Right column shows the speeds in reverse direction. The color bars show the values of the speeds (in cm/s). *x*-Axis is the breadth of the box (in cm) and *y*-axis is length of the box (in cm). Dashed lines represent boundaries of the box and the compartments. HC, Holding Chamber; C1 to C5, compartment numbers 1 to 5. The plots show multiple trials for a single day.

All five rats were implanted with thermocouples to measure respiration frequency during tracking. As shown in Figure 6*a*, rats sniff faster while running toward the odor source (forward sniff rate, 8–10 Hz) compared with running toward the water reward (return sniff rate, 5–7 Hz). This elevated sampling behavior was observed irrespective of whether the rats were running directly or serially toward the target (Fig. 6*b*). We next asked whether high sniffing rates were related to the running speed of the rat. Figures 7 and 8 show instantaneous speeds of the animals during direct and serial tracking, respectively, for a single day. The left column shows forward speeds and the right column shows the return speeds for all 5 rats (*a*–*e*). Large variations in the instantaneous speeds between rats were observed for forward tracking, as well as between direct and serial trajectories. For example, in some direct tracks, rats were slow at the holding chamber, sped up in the middle of the arena, and then slowed down as they approached the odor source. In other direct tracks, rats had near constant (low or high) speeds throughout the run. On the other hand, instantaneous speeds during the return run to water reward was similar in all animals and in both the strategies. Strikingly, in all cases the sampling frequency was higher during the forward run even though the average running speed was lower (Fig. 6*c*). In summary, rapid respiration was associated with the tracking phase of each trial, rather than exertion due to faster running. There was a small difference in running speeds between direct and serial tracking, but the return run was always significantly faster.

### Direct and serial trials differ due to early decision-making

Because in our setup plumes converge near the holding chamber (∼80 cm; Figs. 1*h*, 2*c*), errors in route selection are possible if rats select targets using plume information near the holding chamber and move upwind. Such behavior has been observed in *Drosophila* (Breugel and van Dickinson, 2014) where flies use early cues to surge upwind, followed by a later casting phase upon loss of contact with odor.

**Figure 9.**
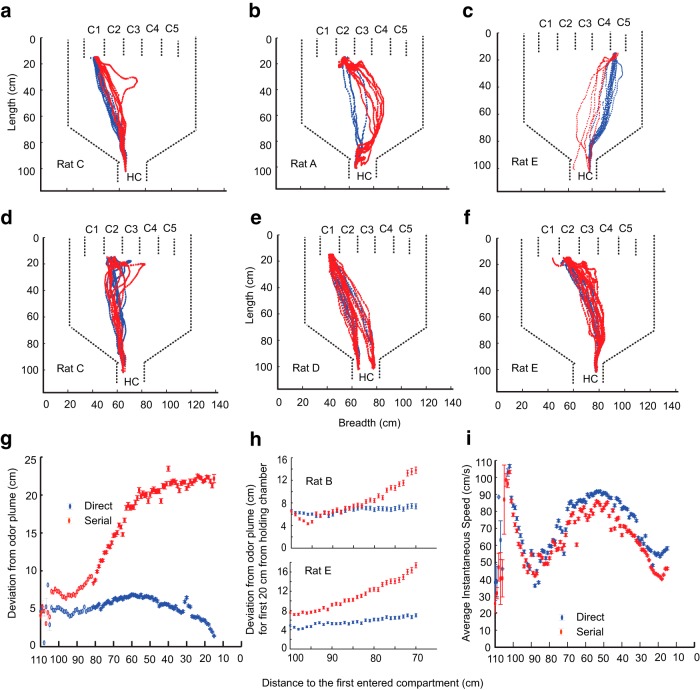
**Serial and direct tracks diverge early after holding chamber**. Tracks until the first entry shown for different compartments **(*a***, ***e***, C1**; *b*, *d***, ***f***, C2**; *c***, C5) in direct (blue) and serial (red) trials. ***a*–*c***, Non-overlapping tracks, whereas ***d***–***f*** show overlapping tracks for both direct and serial trials. First entries in direct trials are to the correct odor compartment, whereas first entries in the serial trials are to the incorrect compartment. Each panel shows tracks from a single session of an example rat as indicated in the plot. ***g***, Deviation from odor plume (cm) as a function of distance from first entered compartment (cm) averaged over all rats, shown for both direct (blue) and serial (red) trials. ***h***, Example data from two rats showing deviation from odor plume in the initial 20 cm from holding chamber. Rat B shows divergence of deviation for direct and serial trials after the 70 cm mark, where as all other rats show divergence from the beginning of the holding chamber (100 cm). ***i***, Instantaneous speed averaged over all rats for every 1 cm toward first compartment entered. Higher speed at 110 cm indicates turning of the animals from water port. At ∼70 cm, the direct and serial speeds begin to diverge from each other.

We first asked whether there was a difference between serial and direct trials that ended in the same compartment. We reasoned that if the movement was odor guided in direct but not serial trials, then the trajectories should differ. We found that overall trajectories to the same compartment could be either non-overlapping (example, 1 d session; Fig. 9*a*–*c*) or overlapping (Fig. 9*d*–*f*) indicating that target routes are not necessarily fixed. A similar variability was seen in the initial (>80cm) part of the track, which was highly overlapping in some cases but different in others (Fig. 9*a*–*f*).

We then checked if errors leading to serial trials were evident at the outset of the track. We computed the distance from the rat track to the odor plume in direct and serial trials respectively (see Materials and Methods). On average rats already showed ∼2 cm greater deviation in serial trials even very close to the holding chamber (Fig. 9*g*). Except for Rat B (Fig. 9*h*, top), all other rats start out further from the odor plume in serial trials (Fig. 9*h*).

As a final comparison of direct and serial trajectories, we compared the instantaneous speed profile for direct and serial trials. We found that the average instantaneous speed profile (see Materials and Methods) diverged slightly between serial and direct trials after ∼70 cm along the length of the box (Fig. 9*i*; *p* < 0.01, paired *t* test). Overall, these comparisons suggest that serial and direct trials are different near the holding chamber and diverge further as they approach the selected compartment.

### Rats can generalize odor tracking to novel odors

**Figure 10.**
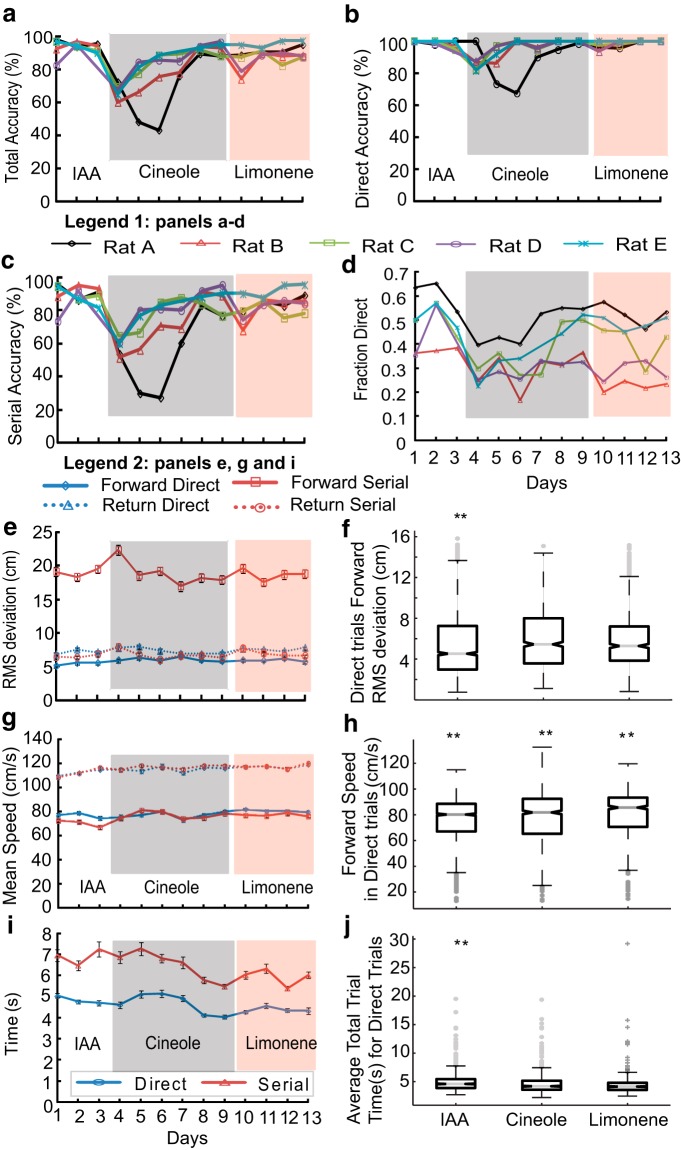
**Rats rapidly learn to track novel odors**. ***a***–***d***, ***e***, ***g***, ***i***, The white region represents baseline (IAA) odor delivery; gray region represents Cineole, and red represents Limonene. ***a***, Total tracking accuracy. There is a major drop on introduction of cineole, a smaller one for limonene. ***b***, Success rate in direct trials. There is a drop only in the first few days with cineole. ***c***, Similar plot as ***b*** for serial trials. There is a big drop at the start of cineole. ***d***, Fraction of trials using direct tracking for all rats. This drops toward chance during the initial few days with cineole. ***e***, Average RMS deviation across all days for all rats. There is remarkably little increase for forward direct trials. ***f***, Whisker box plot of RMS deviation in forward direction for direct trials. Data is pooled for all rats. *N*d (IAA) = 1851, *N*d (Cineole) = 921, *N*d (Limonene) = 724. RMS values of days for Cineole and Limonene are significantly different from IAA but not from each other (**p < 10^−10^, KW Tukey HSD test). Gray line is the median. Lower and upper edges of the box represent 25^th^ and 75^th^ percentile. Whiskers represent the extreme data points and gray crosses are the outliers. ***g***, Mean forward and return speeds for direct (blue) and serial (red) trials for all rats in the forward (solid lines) and return (dashed lines) direction. ***h***, Whisker bar plot of the average mean speed for direct trials combined for days with IAA, Cineole and Limonene as tracking odor. All speeds are significantly different from each other (**p < 10^−10^, KW Tukey HSD test).Note that the speed distributions are positively skewed so the medians in the whisker plots are higher than the means from ***g***. ***i***, Total trial time averaged for all rats for direct (blue) and serial (red) trials. ***j***, Whisker box plot for total trial times in direct tracking for IAA, Cineole, and Limonene days. Novel odors were significantly different from IAA, but not from each other (**p < 10^−25^, KW Tukey HSD test). Error bars on all line plots are SEM values.

At this stage, we had characterized the basic air-borne odor-guided tracking behavior of the rat in a known arena. In the next set of experiments, we examined a range of perturbations to assess the robustness of the behavior. We first asked whether the rats could track odors other than the one they were trained on. When presented with novel odor (Cineole) for the first time, tracking accuracy initially dropped, followed by a recovery period over 4 d (Fig. 10*a*). A second novel odor presentation (Limonene) took a much shorter time (1 d; Fig. 10*a*) to locate. Both direct and serial tracking accuracies were affected with a larger effect on serial tracking (Fig. 10*b*,*c*). The fraction of trials where animals took a direct path varied between the five rats (Fig. 10*d*), though in the case of cineole, the fraction of direct trials decreased in each module, followed by an increase as the learning progressed. To observe the effect of different conditions on tracking, we focused on RMS, speed, and time for direct trials. The dependence of these parameters on training is shown in Figure 10 *e*, *g*, and *i*, respectively. There was a small but significant increase in RMS values when data were pooled for all days of a given module, (Fig. 10*f*; **p < 10^−10^, KW Tukey HSD test) with introduction of cineole (6.01 ± 0.1 cm, *N*d = 921) and limonene (5.9 ± 0.1 cm, *N*d = 724). The distributions of speeds across days for cineole and limonene were significantly different than baseline (Fig. 10*g*; *p* < 10^−5^ KW Tukey HSD test), whereas the average speed pooled across all days was lower for cineole (77.5 ± 0.6 cm/s for cineole vs 80.6 ± 0.66 cm/s for limonene; Figure 10*h*; **p < 10^−10^, KW Tukey HSD test). Decrease in total trial time for direct trials was small but significant (4.5 ± 0.05 s for cineole and 4.3 ± 0.05 s for limonene; **p < 10^−25^, KW Tukey HSD test; Fig. 10*i*,*j*) compared with the baseline trials. Thus, rats were able to learn and generalize the odor-tracking task to different odors, over a few days. Although there were changes in RMS deviation from the odor track, and also in the speed of the direct trials, these were quite small.

### Unilateral nostril occlusion has varying effects on trial time, but not on accuracy

**Figure 11.**
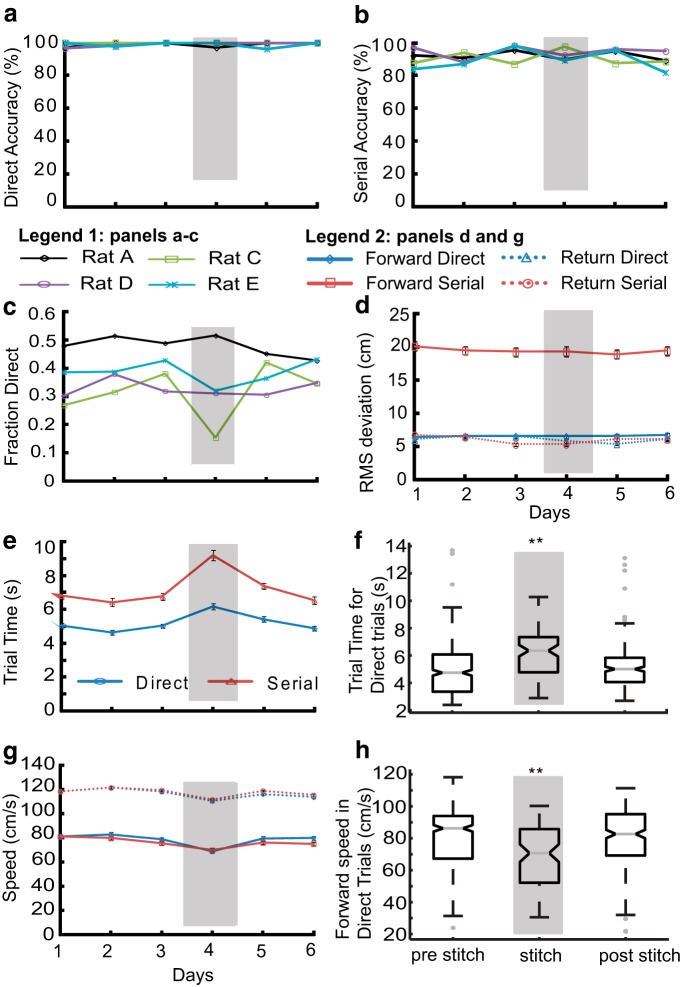
**Nostril stitch does not affect accuracy**. Gray shaded area represents the day of unilateral stitch. ***a***, Percentage of correct Direct trials out of total number of Direct trials (no effect on accuracy, *p* > 0.05, Student’s *t* test). ***b***, Percentage of correct serial trials out of total number of serial trials. ***c***, Fraction direct trials. ***d***, Averaged RMS deviation pooled for all rats for direct (blue) and serial (red) trials in forward (solid lines) and reverse (dashed lines) direction. No significant differences were observed for RMS values (data not shown). ***e***, Total trial time for all rats for direct trials (blue) and serial (red) trials. ***f***, Total time for direct trials pooled for all rats for three groups: pre-nostril stitch (pre-stitch, *N* = 425), nostril stitch (stitch, *N*=92), and post-nostril stitch (post-stitch, *N* = 243). Total trial time for stitch days are significantly higher than pre and post-stitch days (**p < 10^−10^, KW Tukey HSD test). ***g***, Mean forward and return speeds for all rats. ***h***, Mean speeds for direct trials in forward direction. Stitch day speeds were significantly lower than pre- and post-stitch days (**p < 10^−7^, KW Tukey HSD test). Error bars on all line plots are SEM.

Rats use bilateral stereo input to achieve higher accuracy in odor-source localization as well as surface-borne odor tracking (Rajan et al., 2006; Porter et al., 2007; Khan et al., 2012; Catania, 2013). We tested rats with unilateral nostril block in our air-borne odor-source localization task with Limonene as the tracking odor. Remarkably, we observed no differences in accuracies (Fig. 11*a*,*b*) or averaged direct trial RMS deviation (Fig. 11*d*) pooled across all rats. Barring one rat (Fig. 11*c*), the fraction of direct trials was also consistent across days. Small differences were observed in speeds and trial times of rats on the day of the nostril stitch. There was a significant increase (**p < 10^−10^, KW Tukey HSD; Fig. 11*e*,*f*) in the time taken to finish direct trials during stitch days (*N*d = 91, mean = 6.17 ± 0.17 s) compared with pre- (*N*d = 425, mean = 4.89 ± 0.08 s) and post-stitch (*N*d = 243, mean = 5.12 ± 0.1 s) days. Similarly, forward speeds for direct trials were also slower (**p < 10^−7^, KW Tukey HSD test; Fig. 11*g*,*h*) during the stitch days (69.2.3 ± 1.9 cm/s compared with ∼80 cm/s for both pre- and post-stitch days). Thus, animals maintained high accuracy in tracking despite loss of stereo information, at the expense of small increases in trial time and decreased forward speeds.

### Identity of background odor determines tracking accuracy

**Figure 12.**
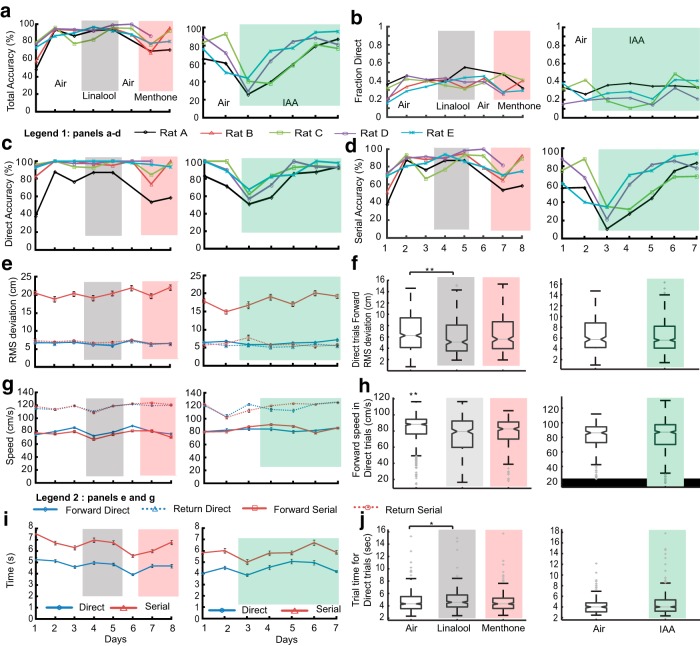
**Background odor identity transiently affects accuracy of odor localization.** Foreground tracking odor is Limonene (0.5%). Control (air background) is represented in white. Rats take 1-2 days to stabilize to the reduced tracking odor concentrations. Linalool background is gray shaded, Menthone is red shaded and IAA is green shaded. ***a***, Total accuracy of all rats with background odors. There is a small drop for menthone, and a large drop for IAA. ***b***, Fraction of direct trials for all rats. ***c***, Accuracy of direct (***d***) and serial trials for all rats with different background odors. There is a particularly large dip for IAA. ***e***, Average RMS deviation of all rats for direct and serial trials in forward and return direction. ***f***, Averaged RMS deviation in direct trials during forward tracking. Data pooled for all rats and trials. RMS deviation with air (*N*d = 524) was significantly different from days with Linalool (*N*d = 295), but not from days with menthone (*N*d = 241) as background (**p < 10^−3^, KW Tukey HSD test). RMS deviations for days with IAA (*N*d = 540) did not differ from air (*N*d = 211, KW Tukey HSD test). ***g***, Mean forward and return speeds for direct and serial trials for different background odors. ***h***, Averaged speeds for all direct trials during forward tracking with Linalool, Menthone and IAA as background odors. Speeds for both Linalool and Menthone were significantly lower than baseline (**p <10^−8^, KW Tukey HSD test). Speed for IAA (all days pooled) and air as background were not significantly different. ***i***, Total trial time taken for different background odors in direct and serial trials. ***j***, Average trial time for direct trials pooled across all days for air/Linalool/menthone and air/IAA as background. Air background trial time is lower than Linalool (*p = 0.03, KW Tukey HSD test). Menthone as background did not affect trial time. Average trial time pooled for days with IAA as background was not significantly different from with background air (5% significance, KW Tukey HSD test). Error bars on all panels represent SEM values.

In natural environments, animals have to locate odor direction in the context of many different background odors. To test the effect of odor background on tracking, we introduced background odors into all of the five compartments. To keep the total odor concentration in the system at 1%, our tracking odors were at 0.5% saturation at 0.5 l/min, and so was the background odor (see Materials and Methods). On separate days, we introduced background odors linalool, and menthone (unfamiliar odors) and IAA (familiar odor). In all these cases, the animals were tasked to locate limonene coming from a single compartment. We started these experiments with the tracking odor (limonene at 0.5% saturation) in an air background. This reduced concentration led to an initial changed baseline for multiple behavioral parameters, including accuracy and fraction of direct trials. These rapidly returned to baseline (Fig. 12). We observed that different background odors had different effects on tracking accuracy, but the animals soon recovered. For example, the presence of background linalool (a terpene alcohol) had no visible effect on any of the parameters like total, direct, and serial accuracy (Fig. 12*a*,*c*,*d*, grey shaded region). Background menthone (which belongs to the same family of cyclic terpenes as the tracking odor limonene) had a modest effect on total, direct, and serial accuracies (Fig. 12*a*,*c*,*d*, red shaded region).

In a separate block of sessions, we again introduced limonene at 0.5% in an air background, followed by background IAA, which had previously been used as a reward odor. IAA had a strong effect (Fig. 12*a*,*c*,*d*, green shaded region). Our interpretation is that rats identified IAA coming from each compartment as a potential reward odor. They thus were not able to discriminate between odor targets, initially reducing their tracking accuracies to chance (20%). This outcome serves as an additional control to show that rats were indeed relying on odor to carry out their compartment selection.

The effects of background on other measures of performance were small or absent. Fraction direct was unchanged in all but one rat (Fig. 12*b*). RMS deviation from new baseline (6.9 ± 0.14 cm, *N*d = 524) for direct trials decreased only with linalool (mean = 6.0 ± 0.18 cm, *N*d = 295; Fig. 12*e*,*f*; **p < 10^−3^, KW Tukey HSD test) as background, but not menthone (mean = 6.4 ± 0.2 cm, *N*d = 241). No differences were observed with pooled IAA background (mean = 6.4 ± 0.1, *N*d = 540) as well. We observed decreased speed (Fig. 12*g*,*h*) and increased time (Fig. 12*i*,*j*) with linalool (75 ± 1.1 cm/s) and menthone (77 ± 1.2 cm/s) compared with the new baseline (82.7± 0.8 cm/s, **p < 10^−8^, KW Tukey HSD test). Again, no such differences were observed with IAA as background (83.1 ± 0.8 cm/s). In summary, background odors did have an initial impact on rat tracking behavior but with learning rats were able to discriminate between the background and foreground, and accurately track the relevant odor.

### Increased turbulence was ineffective in changing tracking behavior

**Figure 13.**
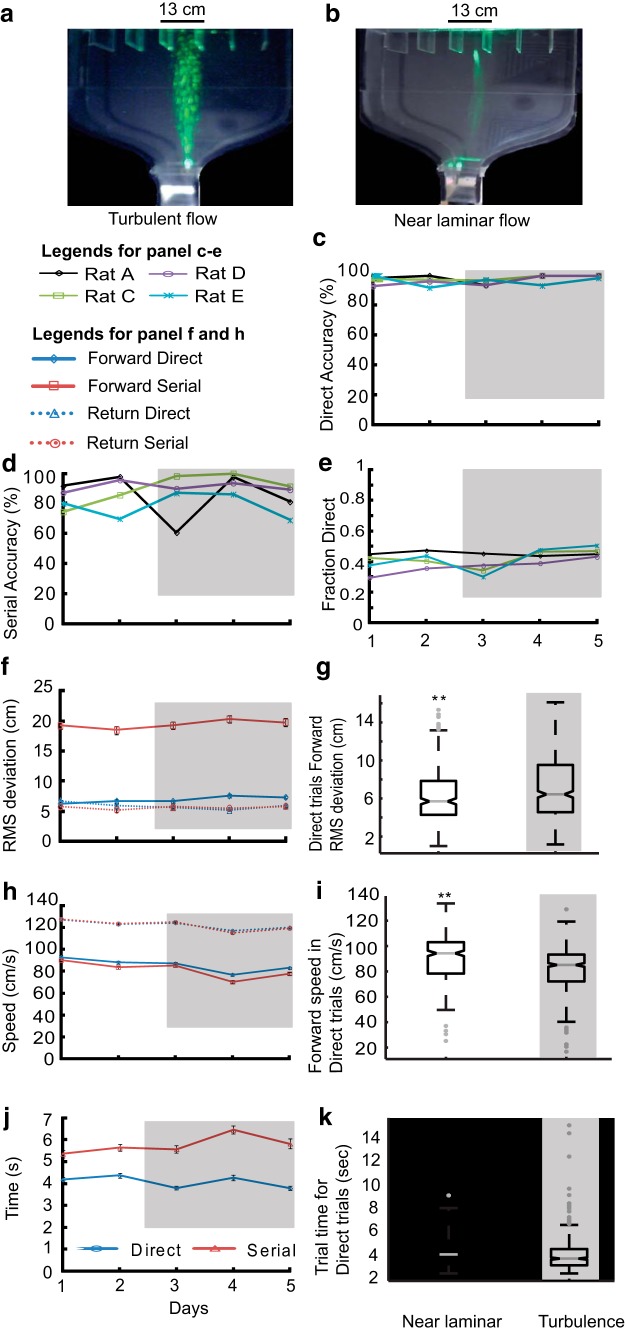
**Increased turbulence does not affect tracking accuracy**. ***a***, Image of box showing turbulent and (***b***) laminar flows, using smoke plumes illuminated by a laser light sheet with the source near the holding chamber. ***c*–*k***, Gray shaded areas indicate days when turbulence was introduced. ***c***, Accuracy of Direct trials and (***d***) serial trials. ***e***, Fraction of direct trials out of total trials. ***f***, Averaged RMS deviation for all rats. ***g***, Whisker bar plot of average speed for direct trials in forward direction for days with and without turbulence (*N*d laminar = 325, *N*d turbulence = 522). The RMS values are significantly different from each other (**p < 10^−3^, KW Tukey HSD test). ***h***, Forward and return speeds for direct and serial trials averaged across all rats. ***i***, Whisker bar plot of mean speeds for days with near laminar and increased turbulence. Speed was significantly lower for turbulent days (**p < 10^−14^, KW Tukey HSD test). ***j***, Trial time averaged across all rats for direct and serial trials ***k***, Whisker bar plot of average trial time for direct trials during near laminar and turbulent airflow. Trial time values are significantly different from each other (**p = 10^−3^, KW Tukey HSD test). Error bars for all line plots represent the SEM values.

Natural odor environments are highly variable and frequently turbulent. Studies on effect of increased turbulence have shown reduction in tracking accuracy in blue crabs (Keller and Weissburg, 2004), but no change or an increase in the tracking accuracy of whelks (Ferner and Weissburg, 2005) and crayfish (Kozlowski et al., 2003; Moore et al., 2015). To study the effect of dispersed odor plumes on tracking, we removed the carbon filter near the compartment end, without changing the rate at which the air was suctioned into the box. This resulted in introduction of eddies into the airstream and a broader odor plume (Movie 4; Fig. 13*a*). As the turbulent plume progressed, its width exceeded the width of the compartment and its profile was highly variable in both temporal and spatial dimensions. In our experiment, with the exception of one rat (Rat A), we did not observe any change in accuracy (Fig. 13*c*,*d*) or fraction direct trials (Fig. 13*e*). On the other hand, RMS deviation increased (Fig. 13*f*,*g*; from 6.4 ± 0.16 cm, *N*d = 325, to 7.2 ± 0.14 cm cm, *N*d = 522, **p < 10^−3^). The effect on speed and total time of tracking were small but significant (mean speed = 82 ± 0.7 cm/s; Fig. 13*h*,*i*; **p < 10^−14^; mean time = 3.9 ± 0.7 s, Fig. 13*j*,*k*; **p = 10^−3^, KW Tukey HSD test). We looked into the subcategories of direct tracking, and found an increase of offset trials from 6 to 11%. No increase in the fraction of trials with casting was observed (8%). In effect, most rats were readily able to compensate for turbulence in their odor-tracking performance with few modifications to their tracking behavior.

Movie 4. **Turbulent airflow**. Top view of behavior box with turbulent flow conditions. Smoke plumes are released into the box from compartment 4 and visualized using green laser light. Video recorded at 25 Hz. 10.1523/ENEURO.0102-15.2015.video.4

### Sniff-triggered course corrections were not observed

**Figure 14.**
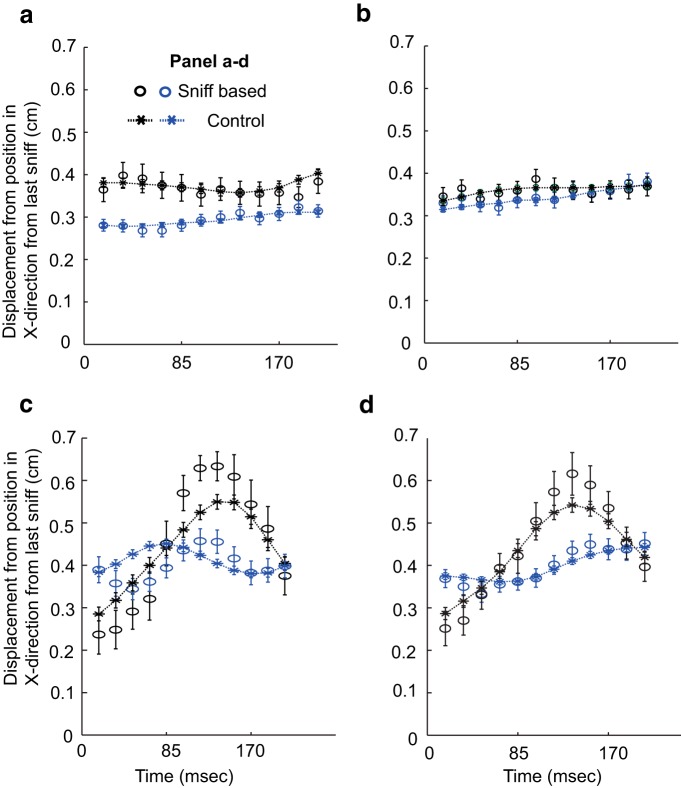
**Trajectory changes are not triggered by odor sampling.** *y*-Axis shows average displacement per frame since last sniff for forward track. *x*-Axis is time (ms) since last sniff. Each data point is a successive frame. For each plot, black and blue represents example data from different days. Star markers with dashed lines show control (see Materials and Methods), while circle markers represent post-sniff (see Materials and Methods) displacement values. Plots ***a*** and ***b*** show baseline tracking data for Rata A and D, respectively. Plot ***c*** shows pre-stitch baseline for Rat D, whereas plot ***d*** shows (Limonene + IAA background) data for Rat D. The control and sniff related displacements were not found to be significantly different from each other in all cases (*Z* test, Bonferroni correction, at 0.001 significance level).

We analyzed if sniffs triggered changes in trajectory during tracking under any of these conditions. To do this, we computed sniff-triggered trajectory histograms and compared these against non-sniff-triggered trajectories (see Materials and Methods). We did not find significant differences between the sniff-triggered and control cases in any of the 46 of 350 recording sessions that cleared sniff-classification criteria (criteria specified in Materials and Methods; data shown until 192 ms post-sniff; Fig. 14*a*–*d*).

In five cases (pre-nostril baseline and IAA background), there were apparent deviations at the 8–10th frame (ie, 137–170 ms post-inhalation; Fig. 14*c*,*d*), but these were not significantly different from the control. If trajectory corrections were sniff related, they would be expected to happen at around this time (Wesson et al., 2008). Thus, the current sniff and tracking data do not support the hypothesis of sniff-triggered changes in tracking trajectories.

### Run-and-scan is more efficient than casting over a wide range of conditions

To integrate these observations, we constructed two models to better understand the tradeoffs between run-and-scan behavior and the familiar casting strategy. In the first model, we used the observations that rats used direct trials ∼50% of the time (Fig. 4*a*) and were correct almost all the time in such cases (Fig. 16*a*). We also used the observation that error trials adjacent to the correct port accounted for ∼25% of the total trials (Fig. 3). We assumed that in such cases the rats immediately corrected themselves, thus wasting only one scan time (tScan). In the remaining 25% of cases, we assumed that the rats never resampled an already visited compartment, and took precisely tScan seconds to visit each. Thus the expected time for a trial was as follows:

$$\begin{array}{c} tRun=D/v+tScan\times (0.25+0.25\times (N-1)/2),\\ \text{which}\;\text{simplifies}\;\text{to}\\ tRun-D/vRun+tScan\times (N+1)/8. \end{array}$$

Here, *tRun* is expectation time to complete the run, *D*, is distance to target(s), *vRun* is running speed, and *N* is number of compartments.

In the second version of this model, the rats still used direct trials 50% of the time. Here, we assumed that if the rats made an error, they serially scanned the remaining compartments including the adjacent ones. We tested this model because only three of five rats showed a significantly higher likelihood than chance of picking a compartment adjacent to the correct one (Fig. 3).We again assumed that the rats never resampled and took the same tScan seconds per compartment. We then obtained the following:

tRun=D/v+tScan×(N−1)/4.

Finally, we assumed that in casting behavior the animals advanced toward the target at fixed speed *vCast*, thus giving the following model:

tCasting=D/vCast

**Figure 15.**
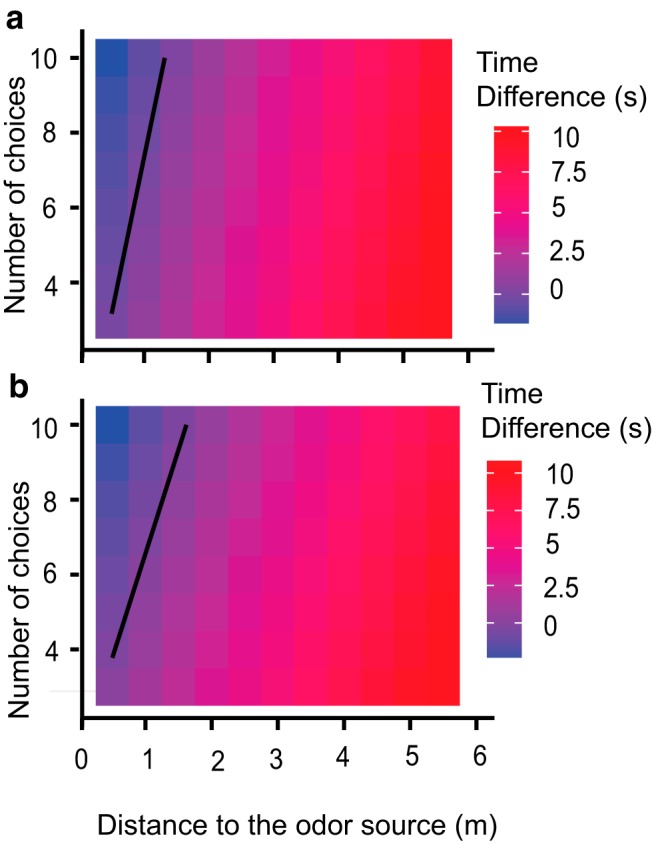
**Run-and-scan behavior is usually faster than casting.** Color maps of time difference between casting and run-and-scan. *x*-Axis shows the distance to the source (meters) and *y*-axis shows the number of possible targets. Casting would be preferable for time difference <0 (blue shaded bins), whereas run-and-scan would be preferable for time difference >0 (red shaded bins). The black line indicates the parameters for which they are equal. ***a***, Model 1, where rats find the correct adjacent compartment on the second try. ***b***, Model 2, where there is no special advantage in finding odors in the adjacent compartment.

In these models, *N* = 5 and *D*∼1 m are known from the configuration of the arena. The running speeds *vRun* for both run and scan were ∼0.8 m/s (Fig. 4*d*). Although we are not aware of direct data for running speeds of rats during free-running casting behavior (*vCast*), previous studies on surface-borne odors suggest that casting may become limited by sniffing rates at around 0.2 m/s (Khan et al., 2012). Here, we assumed that the speed during casting was roughly *vCast* = 0.3 m/s, though the broad conclusions of the model were not very sensitive to this. We estimated *tScan* as 1.5 s, as described in Materials and Methods. We computed run-times for a range of *N* and *D* for each strategy and plotted their difference (Fig. 15). This gave the surprising prediction that run-and-scan was a better strategy in most cases, especially at greater distances. In summary, this model suggests that the run-and-scan strategy is preferable at greater distances in situations where there are known targets, but casting was advantageous for free-range search.

## Discussion

We have found that rats achieve high accuracy and robust performance in tracking air-borne odors in a familiar environment, but do not use casting (zigzag scanning) to do this. Instead, they preferentially attempt a subset of targets and approach them in a rapid, odor-guided, but somewhat error-prone manner. They resort to serial scanning if their initial target selection is incorrect. We suggest that this “run-and-scan” behavior is an alternate to casting in an environment where there are a small number of known targets or potential routes, and may offer advantages in speed and robustness.

Casting is a well established odor-tracking strategy, and has been observed in numerous surface-borne as well as air/water-borne contexts (Vickers, 2000; Porter et al., 2007; Khan et al., 2012). A distinct strategy has been reported for short-range target selection: casting to ascertain gradients, followed by stereo to home in on the target (Catania, 2013). Our results show that, in our specific arena, animals achieve good odor-guided performance but do not rely on casting. Surprisingly, our model suggests that the familiar casting strategy is not as efficient as run-and-scan in most long-range contexts where there is additional target distance information. If we strip away the model terms related to direct trials, run-and-scan wins simply because the initial run saves time, and subsequent scanning is not very expensive, as long as there are not too many possible targets or they are not too far apart. Casting becomes essential when the distance to the odor source is unknown. Extrapolating from the conditions of our arena, we suggest that the key determinants for observing natural run-and-scan behavior would be: (1) known distances or paths to the targets, either through previous experience or through other sensory cues, (2) a few distinct targets rather than a continuum, and (3) upwind odor cues. Although we are not aware of studies that have examined this, we suggest that these conditions may occur frequently in natural contexts. The behavior we observed was quite robust to perturbations. Although the behavioral protocol almost ensured high final-target selection accuracy, our emphasis here was on the choice between direct and serial trials, and the quantitative readouts of tracking during the trials. To first order, it was remarkable how consistent the basic run-and-scan behavior was under a wide range of manipulations. The only exception to this general observation of robustness came when we used a familiar odor (IAA) as background along with the tracking odor. In this case, the animals were simply confused about the identity of the odor that specified reward, and in a few days learned the task in this context as well.

**Figure 16.**
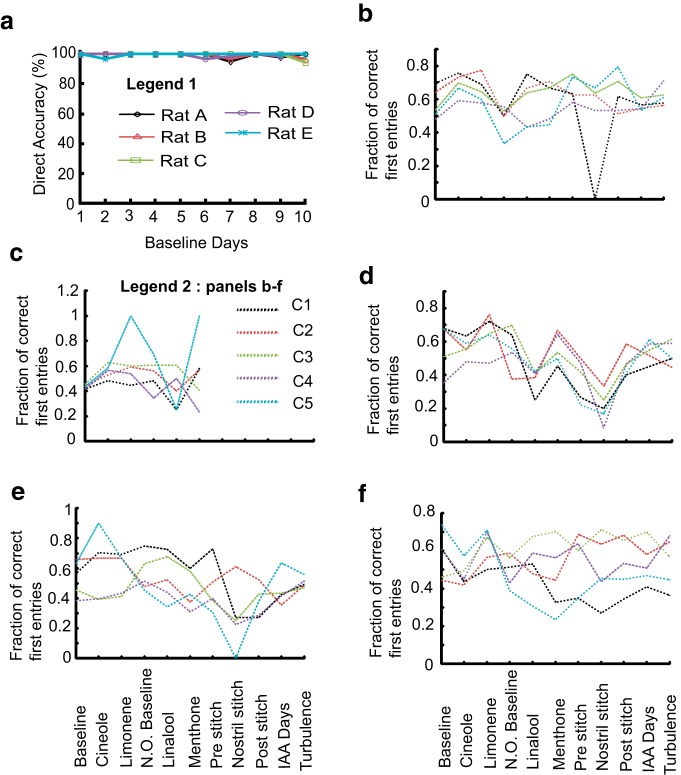
**Fraction Direct performance. *a***, Accuracy of direct trials over 10 d of baseline training for all five rats (Legend 1). ***b***–***f***, Data for all experiment subsets, ie, [baseline–novel odors (Cineole, Limonene)–novel background odor (Linalool, menthone)–pre-stitch–Stitch–post-stitch–IAA background–turbulence] from five rats are shown from ***b*** onward. Each compartment is a color-coded dashed line (Legend 2). The plots show fraction from total entries into a given compartment when that compartment was the odor port, ie, fraction = number of first entries in that compartment when odor was on/total number of first entries in that compartment

When the quantitative parameters of behavior were examined, it was clear that the rats did not randomly pick a target and then ignore odors as they ran: the animals were sampling rapidly for the entire route, and their speed was slower than when they were running back for the reward. In some cases, a last-second course correction was apparent (Fig. 5*d*,*e*, left and center columns). However, unlike in casting behavior, we did not see evidence for sniff-triggered course corrections (Fig. 14). Further, in difficult situations (unilateral nostril block, background odor, or turbulence) there was a small but significant effect on speed. On average, rats correctly made a direct entry into the odorized compartment 40–70% of the time (Fig. 16*b*–*f*). This fraction varied within this range across compartments and with perturbations. We interpret this to mean that the animals did indeed improve performance and fraction of successful direct trials by continual monitoring, and sustained above-chance direct trials despite perturbations.

How efficient is stereo guidance in these conditions? Previous results for air-borne as well as surface-borne odor tracking show a characteristic scanning or casting behavior (Thesen et al., 1993; Vickers, 2000; Porter et al., 2007; Gomez-Marin et al., 2011; Khan et al., 2012). This has been shown to be quite sensitive to stereo sampling (Duistermars et al., 2009; Gomez-Marin et al., 2010; Khan et al., 2012; Catania, 2013). However, target selection has been shown to have multiple phases (Moore et al., 1991; Thesen et al., 1993), including an initial non-stereo-guided phase and subsequent refinement using stereo (Catania, 2013). Here we found that stereo has little effect on odor-source localization for air-borne odors coming from known potential targets. Our results for nostril occlusion are in contrast to surface-borne tracking in animals (Porter et al., 2007; Khan et al., 2012) where increased deviations are observed with unilateral sensor block. One explanation is that in our behavioral setup, air-borne cues are distant in nature, whereas use of bilateral comparison is more effective near the source of the odor, where concentration gradients are steepest (Catania, 2013).

Why do rats persist with serial scanning? In our study, the rats adopted the direct trajectory only 40–70% of the time, even though the direct trials took less time and were as accurate as serially completed trials. Further, additional reinforcement (2× reward) for direct trials did not raise the fraction of direct trials to >70%.

**Figure 17.**
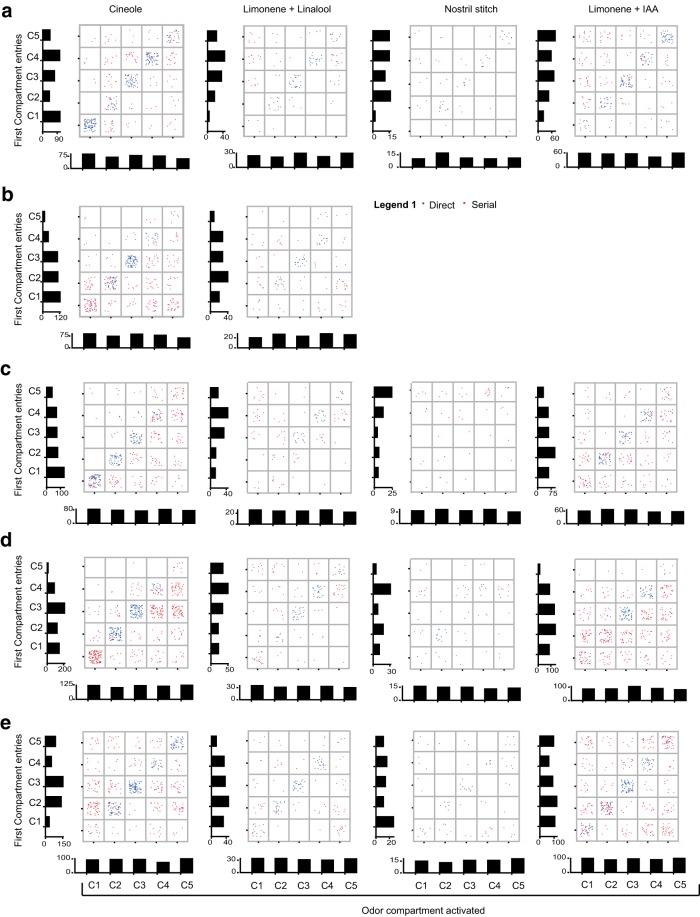
**First compartment entries for different experimental modules. *a***–***e***, Data from five rats. Each column represents an experimental module. Column 1 is days of novel odor (Cineole); Column 2 is days of tracking + novel background odor (Limonene + Linalool); Column 3 is the day of unilateral nostril stitch; and Column 4 is days of tracking + familiar background odor (Limonene + IAA). Serial and direct trials are color coded (Legend 1). *y*-Axis is the number of first entries in each compartment. *x*-Axis is odor compartment activated.

One possible explanation for the persistence of serial trials could be the preference of rats for the side chambers, as rats are known to prefer to move along walls. In contradiction to this hypothesis, we found that the first compartment entry in direct as well as serial trials was in fact biased toward the central compartments (Figs. 3, 17).

Another possibility might be the dichotomy between goal-directed and habitual behavior (Balleine and O’Doherty, 2010; Keramati et al., 2011). In our work, the direct tracking could be interpreted as goal-directed as it is based on immediate assessment and decision-making based on sensory data, and the serial as a habitual tracking where actions could be initiated without deciding on where the target is. However, both serial and direct trials had high sniffing rates, and rats were actually running slower in the serial trials. Thus, it is unlikely that one could classify serial trials as other than goal-directed.

From our observation that serial and direct trials differ very early in the track, we suggest that initial trajectory decisions are made early but in an error-prone manner (Fig. 9). Thus, serial tracks result from an incorrect guess, whereas in direct trials the early guess remains on the odor trail. Thus, near-guesses may account for the high incidence (∼46%) of serial trials where the first entry was in the compartment adjacent to the correct one. How might the run-and scan behavior apply in ethological contexts? In one scenario, there may be a small number of traversable tracks leading from the entrance of a burrow to possible food (and odor) targets. When tracking, the animal would run toward its best guess and should it fail would scan through the others. Another manifestation of the scan phase of this behavior may occur when animals forage in a target-rich environment, such as a refuse dump, rapidly sampling one location after another.

We suggest that serial scanning is frequent simply because rats prioritize speed over accuracy in this behavior. Indeed, from the model calculations, the higher-than-chance accuracy of direct trials could be viewed as a small bonus, but not the central advantage of the run-and-scan strategy. Thus, serial scanning should be seen not as inefficient fallback behavior, but rather as the key part of the run-and-scan behavior pattern, which is effective at long distances and when the possible targets are known.

## Synthesis

The decision was a result of the Reviewing Editor Howard Eichenbaum and the peer reviewers coming together and discussing their recommendations until a consensus was reached. A fact-based synthesis statement explaining their decision and outlining what is needed to prepare a revision is listed below. The following reviewers agreed to reveal their identity: Matthieu Louis, Tom S. Collett

The reviewers were generally enthusiastic about this study and feel it provides a valuable systematic description of how rats localize odor sources of considerable use for subsequent studies on neurobiological mechanisms of search behavior. However, several questions and requests for further elaboration of the findings were raised by both reviewers, as specified in their individual comments.

Reviewer #1:

The manuscript is very clearly written. Careful controls are documented to rule out technical artifacts. The methodology section provides a thorough description of the assay, which should promote its use by the rest of the community. The analysis of the behavioral data is thorough, and yet the result section is easily digestible as the main conclusions are summarized at the end of each section. Both the introduction and discussion are concise and to the point. At the end of discussion, I would nonetheless encourage the authors to speculate a little about ethological relevance of serial casting during (foraging) behavior in the wild. Below, I made several suggestions to strengthen the data analysis in a couple of directions.

1) Fig 2e-f: the trajectories suggest that changes in orientation are usually associated with inhalation episodes. Could the authors quantify this potential trend? Based on this, do they find that trials bringing the rat directly to the right port are underlaid by a larger fractions of "correct" changes in orientation compared to the trials leading to serial casting?

2) Fig 9: upon introduction of a new odor in the arena, rats slow down. Is this slowing down coupled to an increase in sniffing frequency?

3) Fig 10: do rats sniff more frequently upon stitching of a nostril? Can the sniffing frequency be viewed as a proxy for the uncertainty level of the stimulus encoding?

4) The authors report that about 25% of the trials bring rats to a port adjacent to the correct port. Could they also quantify the probability that a rat finds the right port right after having picked the adjacent port? Does this probability differ from conditions where the first port sampled is further away from the correct one?

Minor comments:

Line 419: the reference to figure appears to be misplaced.

Line 211: in the main text and figure captions, it would helpful to use either "serial" or "sequential" sampling. Using both terms is prone to confusion.

Reviewer #2:

This interesting paper suggests that in familiar surroundings, in which an odour can occur in one of several discrete nearby positions, rats adopt a strategy in which they approach the source directly without casting and if they make an error and pick the wrong position they reach that position and then move laterally from one possible source position to another. The authors show that this strategy works well with laminar air flow and that it persists under a number of other conditions (turbulence, confusing odours etc.). The research seems to be technically very thorough, though I cannot back this opinion up with first-hand knowledge of the techniques used, and is fully and clearly described.

While the basics of the strategy seem clear, I missed an account of what in detail the strategy might involve. Once the rat has learnt a route, when and what kind of corrections does it make when it loses the odour plume? And second what keeps the rat going up the odour plume once it is in it?

A suggested answer to the last question comes from beautiful research on Drosophila following odour plumes (F. van Breugel and M. H. Dickinson, Current Biology 24, 274-286, 2014). Detecting an odour causes the fly to turn and to surge upwind and also to become sensitised to particular visual features. If rats are similar in moving upwind on detecting a relevant odour they would follow the plume. But if they make decisions too near the narrow tunnel at the start, then flying upwind would result in their attraction to the central compartments whether or not that is where the plume originates. Such an effect might possibly account for paths being biased to the central compartments (Fig. 1a). In Drosophila, the response of flying upwind persists for a short while after the odour is lost thus enabling the fly to follow a plume when turbulence disrupts the plume's continuity. This effect would also explain some of the rat's behaviour in this paper.

Potential information for answering the first questions is in Fig 5, but I found this figure quite hard to intuit and wonder whether it might be easier for a reader if the rat's path were shown as a line superimposed on the odour plume. The findings in Fig. 5 may be highly significant for an understanding of the rat's path and it would be great if the information there could be extended by showing more examples. For instance, does the rat make an initial decision and then follow a route which might depart from the plume without immediate correction because the route is in part visually or motorically driven or because of persistence of the upwind response. Are the corrections made to rejoin the plume real corrections or just the following of a route? If they are corrections is this evident in sniffing patterns and what odour information is available to them when they make corrections? Fig 1h, which shows the divergence of the plumes, suggests that it might be difficult for rats to pick between routes before about 80cm along the box. Does the speed profile and the rat's sinuosity along the path support this possibility? That might be the case for rats in Fig 8a and d (??), but not the others. Are 'a' and 'd' more accurate in their decisions (i.e. relatively fewer serial trials?).

In the serial tracking paths, is the initial approach when the rat goes to the wrong compartment the same as the route the rat takes when that compartment is the correct compartment? I.e., do the rats learn relatively fixed routes? To answer this question, it would be helpful to supply a fig which shows routes for the same animal to the same compartments when that compartment emits odour and is correct or does not emit odour and is wrong.

I think it would help readers to explore such questions in the text.

Other points are related to specific lines of the paper.

53-54 at this point not clear what is meant by a 'high divergence' or 'lateral scanning' strategy.

202 Be helpful to say 'holding and reward chamber'.

244 Data were pooled

290 Probably should say whether these measurements were taken once or several times during the experiments. Does the presence of a moving rat distort the plume in front of the rat?

305 How are these 8% incidents of zigzags distributed along the route? Do they occur when the plume is lost?

345 One other interesting feature of Fig 4 is that for each rat apart from 'b' one of the end compartments is particularly well represented as attracting correct responses while the other end is underrepresented. What's happening? Is there some limit to the angular width of the choice area?

363 Where along the route are deviations most likely?

387 Is sniffing related to (a) decision making at the start and (b) deviations from the plume?

428 Might the unilateral data help provide an answer the question posed above (line 387)

483 Does casting/zigzagging increase with turbulence?

517-518. Perhaps re-phrase: 'In summary, this model suggests that run and scan is preferred..........'

555 Is there evidence to say whether the correction is odour or route driven (e.g sniffing followed by a turn? If outside the plume, might the discovery of no odour lead to visual checking?)

575 Might the serial scanning be a function of the experimental arrangement (see initial

remarks)?
